# Assessment of genomic prediction and genetic gain in multi‑population half-sib families in the perennial grass crop intermediate wheatgrass

**DOI:** 10.1371/journal.pone.0357452

**Published:** 2026-09-02

**Authors:** Prabin Bajgain, Jacob M. Jungers, James A. Anderson

**Affiliations:** 1 USDA-ARS United States Dairy Forage Research Center, Madison, Wisconsin, United States of America; 2 Department of Agronomy and Plant Genetics, University of Minnesota, St. Paul, Minnesota, United States of America; Swedish University of Agricultural Sciences, SWEDEN

## Abstract

The University of Minnesota has been domesticating the perennial forage intermediate wheatgrass (IWG) since 2011 using a combination of conventional methods and modern breeding tools such as genomic selection. Globally, most IWG selection nurseries are spaced-planted individuals of several hundred genotypes whereas commercial fields established for grain production are row-planted panmictic populations. This study evaluated genomic prediction models and estimated genetic gain in yield and agronomic performance of row-planted IWG half-sib families assessed over 3 years and 2 locations, Lamberton and St. Paul, MN, USA. The strongest trait correlation was negative (r = −0.49) between 2023 St. Paul height and 2022 St. Paul seed size. The three St. Paul environments were more similar for plant height and seed size and so were the Lamberton environments yet no specific trend was observed for grain yield. Evaluation of different univariate and multivariate genomic prediction models showed that multivariate models outperformed the best univariate models by 23 percentage points, yet no single multivariate model was the best predictor of all traits. Cross-environment predictions were the best among St. Paul environments and no single environment was the best predictor of the remaining environments. Genetic gain estimates indicated a 20 kg ha^-1^ increase in grain yield and 3 cm reduction in plant height per breeding cycle. While no single model predicted all traits with high accuracy, results obtained in this study suggest that evaluating IWG sibs in row plots followed by genomic trait predictions could lead to desired breeding progress for desired traits.

## Introduction

Intermediate wheatgrass (IWG, *Thinopyrum intermedium* (Host) Barkworth & D.R. Dewey; 2*n* = 6*x* = 42) is a cool-season perennial rhizomatous grass native to central Europe, the Balkans, and Asia Minor [1,2]. It was introduced to North America in the early 1930s and has largely been used as a forage grass to increase productivity of dry and marginal lands [2]. In the late 1980s, IWG was selected for domestication because of its large seeds, desirable agronomic characteristics, and potential to be used in food products [3]. The University of Minnesota (UMN) started breeding IWG in 2011, has completed seven breeding cycles, and has released one commercial variety named ‘MN-Clearwater’ [4]. The UMN breeding program applies a traditional recurrent breeding selection scheme augmented by modern breeding tools such as high-throughput genotyping, genomic selection, and imaging-based phenotyping methods [5,6].

Of many different modern breeding tools implemented by plant breeding programs, genomic selection (GS) has been one of the most popular and widely adopted ones in recent years. It has been reported that the proper implementation of GS-based breeding schemes can improve trait means in breeding populations and boost genetic gain, and lead to development of superior varieties [7,8]. GS approaches can perform reasonably well in populations with less genetic variation, in low-density marker panels, or when traits have lower heritabilities [9–11]. It has also been well documented that GS approaches can be applied across multiple crop species to improve complex agronomic traits to save time and resources [7,12]. In perennial crop breeding programs such as the UMN’s IWG breeding program, GS has made a significant contribution by not only increasing genetic gain by reducing the time required to evaluate selection nurseries over several years, but also by shortening the timeline of variety development and release [13,14].

Different GS strategies have been assessed and implemented by IWG breeding programs. The first study to evaluate the applicability of GS models in IWG breeding used Bayesian univariate models to optimize the number of markers and population size [15]. The same study, which used UMN Cycle 1 population as the GS training population, reported moderate to high model predictive abilities (ranging from 0.46 to 0.67) for seven agronomic and domestication traits. Following this study, several different prediction models such as ridge regression best linear unbiased prediction (rrBLUP), genomic best linear unbiased prediction (GBLUP), and Bayesian models have been evaluated by the UMN IWG breeding programs and programs in Kansas, USA and Utah, USA [5]. Studies published by the UMN breeding program have reported an increase in prediction by up to 14 percentage points when genetic loci significantly associated with traits of interest were used as cofactors in prediction models [16]. A more recent study that included parents of the training population in prediction models improved trait prediction by up to 11 percentage points [17]. The UMN breeding program also reported that a multivariate model that takes genotype by environment interaction (GxE) effect into account can boost trait predictions by up to 23 percentage points [18].

All GS strategies in IWG so far have relied on genotype and phenotype data generated from spaced-planted selection nurseries. A spaced plant nursery, which is a diverse population comprising of plant genotypes grown at a set distance from one another, is typically preferred in experiments favoring genetic data analysis as the genetic makeup of the nursery is more easily tracked, there is four-times the more genetic variance in the population relative to half-sib populations [19], and are also easy to manage over multiple growing seasons. Yet, both the elite breeding populations and naturally occurring IWG populations are heterogeneous populations because of obligate outcrossing among the plants [20]. One example of these populations is half-sib families (HSFs) where the progeny shares one parent (typically the mother) and have different and mostly unknown, randomly pollinating father plants. Commercially available IWG seeds sold to and grown by farmers are HSFs. This difference in population types between the fields of a breeder and a farmer has made it challenging to translate genetic improvements observed in research plots to commercial grain production acreage.

Trait performance gains measured from elite genotypes in a spaced plant nursery do not directly translate to performance gains observed by farmers in production fields who establish their crops in rows or swards. Breeder evaluations made on rows of half-sib families could reduce this performance gap. For instance, were breeders to also include a row-based experimental design as part of their breeding scheme where advanced germplasm are evaluated and selected in swards that mimic commercial plantings, more similarities in crop performance data between the fields of a breeder and a farmer could be attained. One example of such design is evaluation of sib-families under different treatment regimes and environmental conditions. Studies conducted in other out-crossing perennial forages where sib-families were evaluated for important traits have reported potential in trait improvement through GS-infused breeding approaches [21–23]. Similar adoption by IWG breeding programs could particularly be useful in better understanding complex traits such as grain yield as well as grain yield decline as the plants age [24]. Yet, it is unknown whether GS models perform well with data generated from row plots.

The objective of this study was therefore to assess different genomic prediction models in predicting trait performance of half-sib families established in row-plots and their potential usefulness in an IWG breeding program. Towards this, we evaluated univariate and multivariate models and also applied the models to measure their cross-environmental prediction abilities.

## Materials and Methods

### Germplasm

A population of 208 HSFs was used to conduct this study. These HSFs have been described in detail in our previous study that characterized genomic loci associated with important agronomic traits [25]. Briefly, the maternal genotypes of the HSFs were a subset of the fourth recurrent selection cycle (UMN-C4) from the University of Minnesota’s IWG breeding and domestication program. Establishment and evaluation of UMN-C4 has been described in our previously published study [26]. The 208 mothers were selected out of 637 individuals because they were the best individuals in the UMN-C4 population in the 2019 St. Paul environment for the traits shatter resistance, free threshing ability, grain yield, and seed size. Trait data used to select the maternal genotypes, and subsequently the HSFs, is presented in S1 Table. Note: While the genotype and phenotype data used in this study were published in our previous study [25], this study focuses on genomic prediction whereas the previous study focused on genome-wide association mapping. The objectives, methods, and results presented in the two studies are therefore strikingly unique and different.

### Field experiment

The HSFs were established at two University of Minnesota Research and Outreach Centers: St. Paul (44° 59’ 31.48” N, 93° 11’ 9.27” W) and Lamberton (44° 14’ 33.68” N, 95° 18’ 29.87” W). Two replications of the population were established in fall of 2020 at each location and were maintained for three years in St. Paul and two years in Lamberton (third year discarded due to field management issues). At both locations, the IWG variety MN-Clearwater and an experimental variety candidate MN1502 were planted as repeated checks [4]. All entries were established in 2.3 m (8 ft) long rows with an inter-row distance of 0.3 m (1 ft). A Hege 1000 precision single cone planter (Hege Equipment Inc., Colwich, KS, USA) was used to establish rows by direct-seeding IWG seeds at an approximate rate of 17 kg ha^-1^ (15 lb ac^-1^) at a target depth of 2 cm. In late April of each growing season at each location, the plots were fertilized with nitrogen fertilizer (urea) at a rate of 50 kg ha^−1^. Weed control was primarily done by applying the herbicide Dual II Magnum (S-Metolachlor 82.4%, Syngenta) at a rate of 1.2 L ha^-1^ in late April of each year. After the grain was harvested from the plots in mid-late August each year, plant stands were mechanically mowed to a residual height of 15–20 cm. These management practices are mostly reflective of those adopted by farmers except for application of herbicide for weed control which could vary depending on specific farming practices.

### Genotyping and marker relationship

Genotype data for the HSFs was obtained by genotyping their maternal parents. A genotyping by sequencing approach described by Poland et al., [27] was implemented for this purpose. Briefly, the BioSprint 96 DNA Plant Kit (QIAGEN, Valencia, CA) was used to extract genomic DNA from 10–15 cm long leaf tissue of each mother plant. Double-digested sequencing libraries were created by cutting the genome with the restriction enzymes PstI and MspI and were sequenced on Illumina’s Novaseq 6000 at the University of Minnesota Genomics Center (St. Paul, MN, USA). Obtained reads were filtered for a minimum quality score (Q) of 30, de-multiplexed, and aligned to the IWG reference genome v3.1 [28] using Burrows Wheeler Aligner 0.7.5a [29]. Single nucleotide polymorphisms (SNPs) were discovered on the aligned read stacks using Samtools 1.6 and Bcftools 1.6 [30]. The SNPs were filtered to remove those with minimum minor allele frequency < 3% and missing data (i.e., allelic proportion) of ≤ 10%. Missing data in the final set of 31,640 genome-wide SNPs was imputed with the LD-kNNi method using 30 nearest neighbors in Tassel 5.2.94 [31,32]. To assess relationships among the genotypes, an additive genetic matrix was estimated in Tassel 5.2.94 using the ‘Centered_IBS’ method with default parameters.

### Measurement and analysis of trait data

Three important traits were measured in this HSF population: plant height, grain yield, and seed size measured as thousand kernel weight, TKW. The population was evaluated for these traits in St. Paul in 2021, 2022, and 2023; and Lamberton in 2021 and 2022. Approximately 7 d before the mature plants were harvested, plant height (cm) was assessed by measuring the length of the tallest tiller from the ground up to the spike tip. Measurements were made at two randomly selected spots within each row, away from the edges. To measure grain yield (g), all mature plants within a 0.61 m (2 ft) long section of the row away from the edges were harvested at physiological maturity. Seed heads were separated from the harvested plants and dried in a 36 ℃ oven for 72 h prior to being threshed in a Wintersteiger LD 350 (Wintersteiger Inc, Salt Lake City, UT, USA). The separated grain was weighed to obtain yield per row in g and converted to kg ha^-1^ following the guidelines outlined in the USDA Natural Resources Conversation Service’s Field Office Technical Guide [33]. Threshed grain was sorted to separate de-hulled seeds, which were scanned on a Marvin Seed Analyzer (MARViTECH GmbH, Wittenburg, Germany) to obtain seed size (TKW, g).

Trait data were passed through a mixed model in the R package ‘lme4’ to obtain best linear unbiased estimates (BLUEs) [34]. Particularly, BLUEs for each HSF were obtained by treating each environment (i.e., location and year combination) as a fixed effect in the model while genotypes and replications were random effects. This was followed by removal of the fixed effect for each trait in each environment to correct for trial (environmental) variability. Adjustment for potential spatial correction was not conducted. Outliers among the BLUEs were identified using the function *studres* in the R package ‘MASS’ [35]. Outliers were declared if the absolute studentized residuals in the data distribution were greater than three. Outliers were changed to missing values (NA) after which the BLUEs were averaged. The mean BLUEs were used in all subsequent analyses including data visualization and genomic prediction. Phenotypic data is available in S2 Table.

### Genomic prediction

Trait prediction in this population was done with a few different model types to assess the overall model superiority. These models can broadly be grouped into two types: univariate, i.e., single-trait and single-environment, and multivariate, i.e., multi-trait and/or multi-environment. In the first step, a single environment, single trait model of the following nature was assessed:

### Single trait models

Univariate trait models, while generally considered to be underperforming relative to multivariate models, were assessed to simply understand their applicability as such models have never been trained with IWG HSFs data. Of the most commonly used univariate models in plant breeding are i) rrBLUP, ii) GBLUP, and iii) Bayesian ridge regression (BRR). All three models have been demonstrated to provide sound predictions in specific combinations of crop, traits, and other factors that include genetic and environmental parameters [36,37].

### Multi-environment and multi-trait models

A few multivariate models were used to evaluate any potential improvement in genomic prediction over univariate models. Towards this, three model types were assessed: 1) a multi-trait model (MTM), 2) a multi-environment model (ME), and 3) a multi-trait-multi-environment model (MTME).

Both univariate and multivariate models were executed within the R environment 4.4.2 [38]. The R packages ‘rrBLUP’, ‘sommer’, and BGLR’ were used to run the univariate models rrBLUP, GBLUP, and BRR, respectively [39–41]. The multivariate models MTM, ME, and BMTME were implemented in the R packages ‘MTM’, ‘BGGE’, and ‘BMTME’, respectively [42–44]. More detailed descriptions of model development, operation, parameters, and interpretation of model outcome can be found within the studies referenced above.

### Prediction scenarios

Model prediction ability (*r*) was the Pearson’s correlation value between model-estimated GEBVs of the masked validation set and the training population. Two model cross-validation schemes were implemented to assess model performance [45]:

a. CV2, prediction for genotypes evaluated in at least one environment
b. CV0, prediction of trait values for genotypes in new environment

For all CV schemes, a random four-fold partitioning of the dataset was carried out in 100 replications [46]. In other words, a random and non-repeating 75% of the HSFs was used as the training population and the remaining 25% was used as the validation set.

### Genetic gain from prediction models

Expected genetic gain (ΔG) for the three traits was estimated using the method outlined by Heffner et al. [47] and Rutkoski [48]:


$$\begin{array}{c} {\Delta \text{G\textbackslash{},=\textbackslash{},ir}\sigma }_{\text{A}} \end{array}$$

where *i* is selection intensity, i.e., trait selection differential expressed in units of phenotypic standard deviation (σ_P_); *r* is the predictive ability obtained from genomic prediction models for each trait, and σ_A_ is the square root of additive genetic variance. The highest observed *r* values were used in the equation to maximize ΔG.

## Results

**Fig 1:**
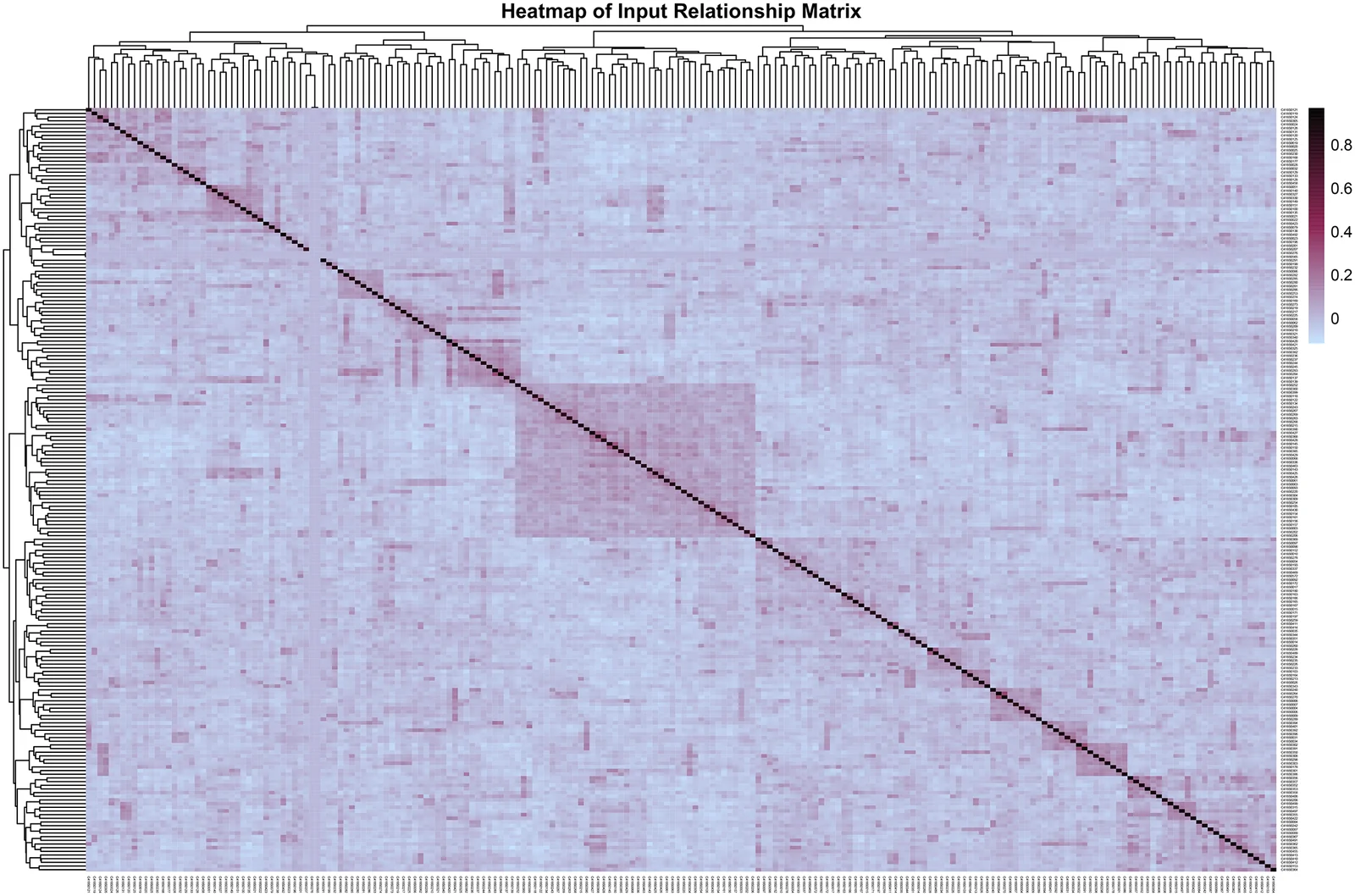
Population relatedness among the HSFs visualized using a heatmap of additive relationship (kinship) values. Genotypes, shown in both axes, are sorted by relationship values where 0 is least related and 1 is most-closely related. The black diagonal represents self-kinship, i.e., value of 1.0.

An additive genetic relationship matrix for the population was visualized as a heatmap to assess population relatedness (Fig 1). No strong population blocking or substructuring were observed across the population. Of all possible pairwise relationships, 49.8% pairs had kinship values lower than the population average value of 0.62. The matrix revealed a weak level of population stratification as the pairwise genetic relationship among most maternal genotypes were low.

**Fig 2:**
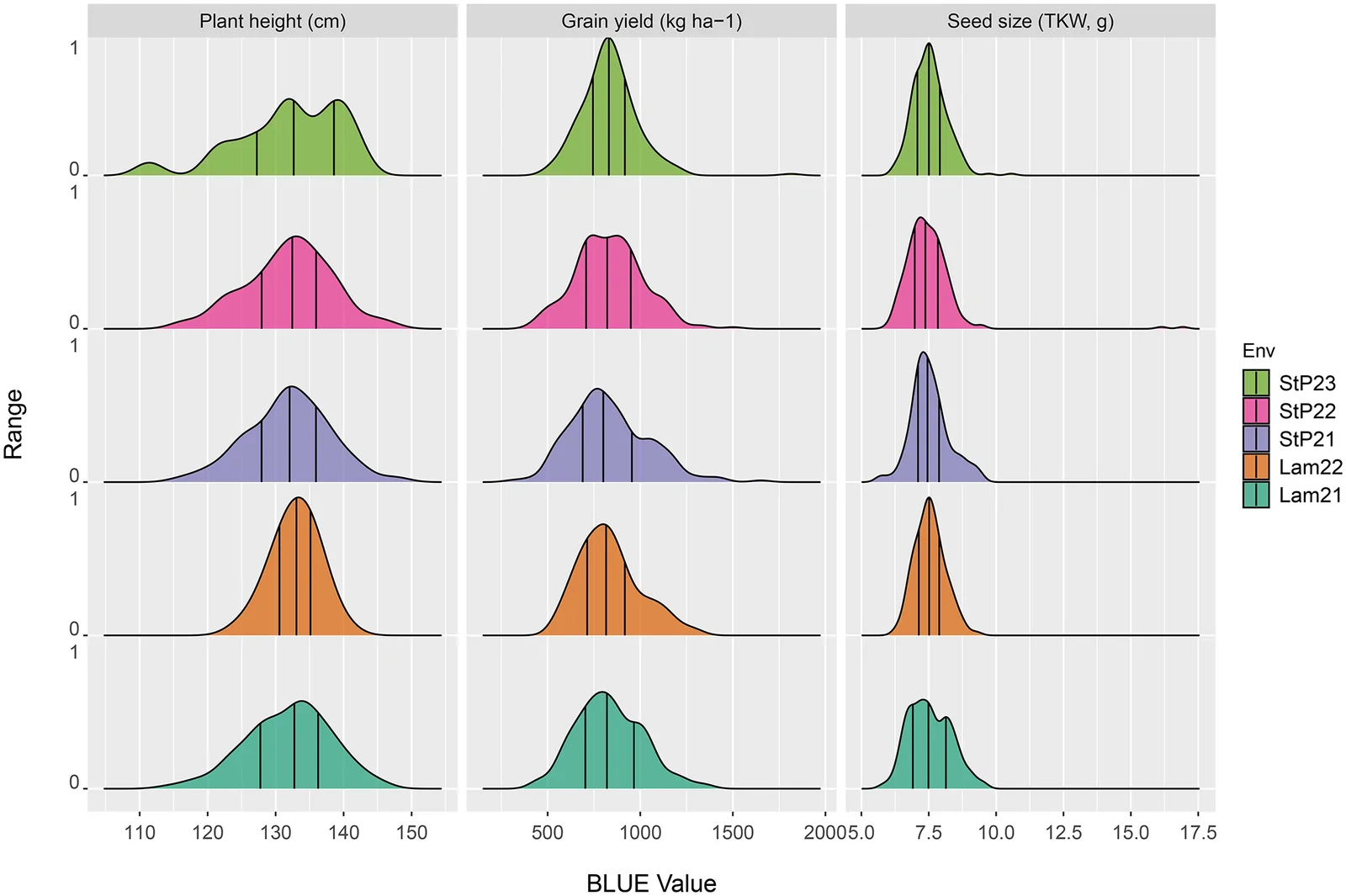
A density ridgeline plot of best linear unbiased estimates (BLUEs) for the three traits plant height, grain yield, and seed size (measured as thousand kernel weight, TKW) in the IWG half-sib population. Traits were measured at two locations in MN, USA during 2021-2023: Lamberton (Lam21 and Lam22), and Saint Paul (StP21, StP22, and StP23). The plot height (y range) within each trait facet is scaled for 0–1 and the vertical lines show the four quantiles of data distribution.

The traits evaluated in this experiment displayed broad quantitative distributions in all environments (Fig 2). The widest distributions for plant height, grain yield, and seed size (TKW) were displayed in the St. Paul 2023, St. Paul 2021, and St. Paul 2022 environments, respectively. Variability in trait expression across the years and locations was also evident from different quantile data (Fig 2) and pairwise trait correlations (Table 1).

**Table 1:** Correlations among the three traits in each environment. The symbols ***, **, and * indicate that the correlation values are significantly different from zero at *α* = 0.001, 0.01, and 0.05, respectively.

		Lam21			Lam22			StP21			StP22			StP23	
		Height	Yield	TKW	Height	Yield	TKW	Height	Yield	TKW	Height	Yield	TKW	Height	Yield
Lam21	Yield	0.15*													
	TKW	−0.02	0.14*												
															
Lam22	Height	0.24*	0.04	0.04											
	Yield	0.10	0.04	−0.04	−0.03										
	TKW	−0.05	0.02	0.01	0.04	0.00									
															
StP21	Height	0.07	−0.06	−0.04	−0.09	0.08	0.05								
	Yield	−0.01	−0.02	0.01	0.00	0.13	0.04	−0.04							
	TKW	−0.08	0.00	−0.07	−0.01	0.01	0.03	0.16*	0.23***						
															
StP22	Height	−0.04	−0.12	−0.05	0.01	−0.10	−0.13	0.16*	0.19**	0.22**					
	Yield	0.10	−0.04	0.08	0.08	0.07	0.02	−0.01	−0.10	−0.05	0.03				
	TKW	−0.12	−0.12	−0.04	−0.03	0.17**	−0.03	0.08	0.20**	0.16*	0.15*	0.00			
															
StP23	Height	−0.03	0.13	−0.10	0.08	−0.35***	0.06	0.23***	−0.27***	0.13	0.21**	0.25***	−0.49***		
	Yield	0.03	−0.07	0.04	−0.02	−0.02	−0.11	−0.14*	0.07	0.04	−0.05	0.11	0.07	−0.19**	
	TKW	−0.07	−0.13	0.00	0.00	−0.01	−0.04	0.04	0.11	0.30***	0.25***	0.08	0.27***	−0.14*	0.21**

To learn the relationships among traits and environments, pairwise trait correlations were calculated for each trait pair in all locations and environments. Results showed a large degree of variation, depending on trial locations and years (Table 1). The strongest correlation was negative (*r* = −0.49), and was observed between plant height in the St. Paul 2023 environment and seed size (TKW) in the St. Paul 2022 environment. The strongest positive correlation (*r* = 0.30) was observed between seed size (TKW) in the St. Paul 2021 and St. Paul 2023 environments. Correlations among the St. Paul environments were overall stronger with the strongest values observed between the 2022 and 2023 St. Paul environments. Between the two Lamberton environments, plant height was strongly correlated (*r* = 0.24) with the rest of the pairwise *r* values ranging from −0.05 to 0.15. The strongest correlation observed among Lamberton and St. Paul environments was negative (*r* = −0.35), and was between plant height in St. Paul 2023 and grain yield in Lamberton 2022.

**Fig 3:**
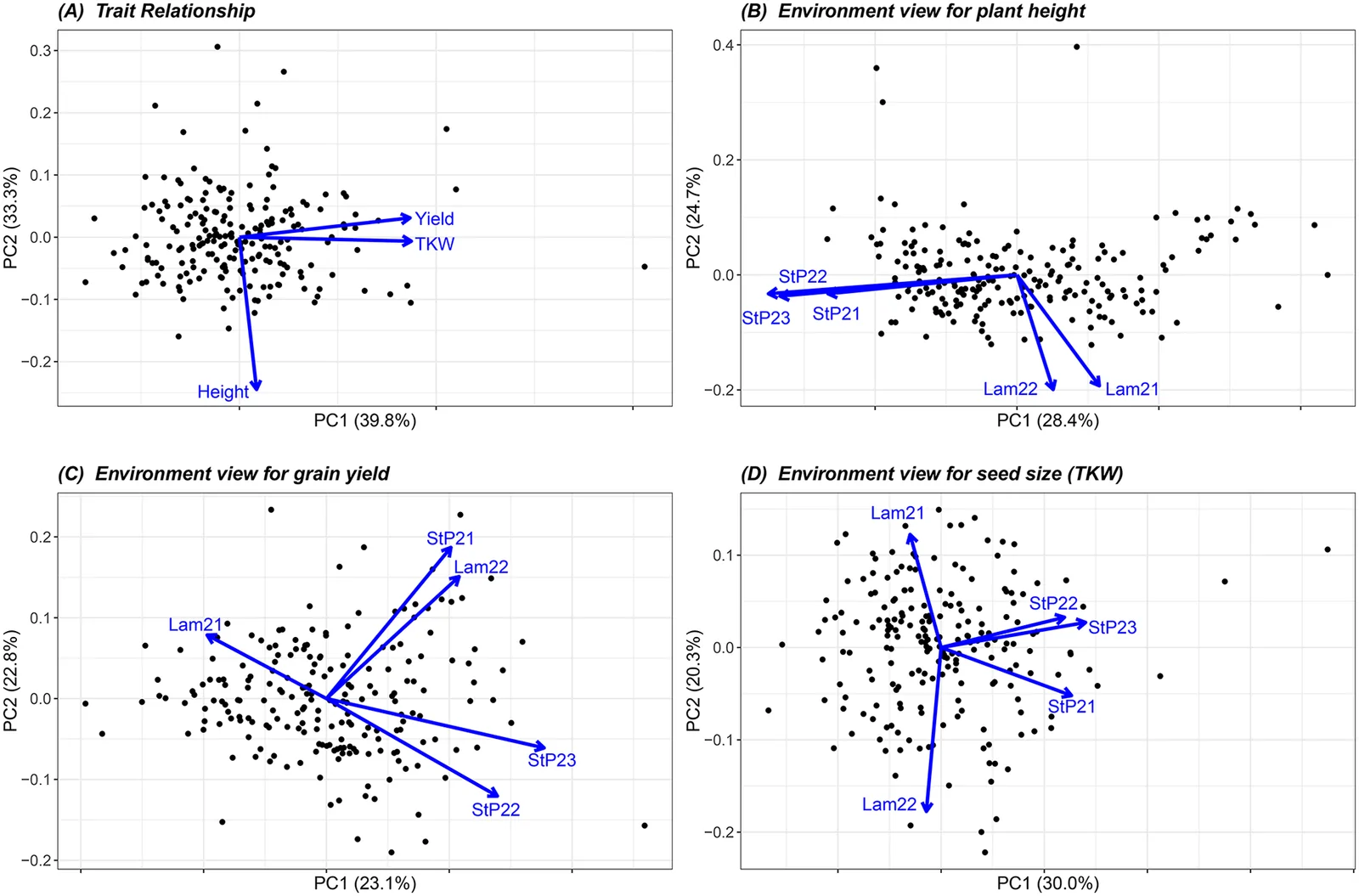
Principal component analysis of the three traits (panel A) and GGE biplots for trait distribution in each environment: plant height (panel B), grain yield (panel C), and seed size (TKW, panel D).

To further understand trends in data distribution, specifically relationships among the environments, trait data were visualized with a principal component (PC) analysis (Fig 3). Distribution of grain yield and seed size (TKW) data indicated a closer relatedness with each other compared to plant height, as it was less related to the other two traits (Fig 3A). For plant height and seed size (TKW), St. Paul environments were more similar and so were the Lamberton environments (Figs 3B, 3D). However no specific trend was observed for grain yield among the five environments (Fig 3C). Yet, the St. Paul 2021 and Lamberton 2022 environments were more similar to each other as also indicated by positive yet insignificant pairwise trait correlations between the two environments (*r* = 0.13, Table 1). Likewise, the 2022 and 2023 St. Paul environments were more similar to each other (*r* = 0.11, Table 1).

Results obtained from training different genomic prediction models revealed that no single model was the best in predicting all three traits (Table 2). Yet, multi-trait and multi-environment models were substantially better than the single-trait and single-environment models. Among the single-trait and single-environment models, the GBLUP model implemented in the ‘sommer’ R package was the best predictor of all traits, i.e., model predictive ability, *r* = 0.22 for plant height, 0.10 for grain yield, and 0.11 for TKW. Among the multi-trait and multi-environment models, the GBLUP MDe model from the R package ‘BGGE’ was the best predictor for all three traits with *r* = 0.45 for plant height, *r* = 0.44 for grain yield, and *r* = 0.25 for TKW. The MDe model therefore improved trait predictions by 23, 35, and 14 percentage points for plant height, grain yield, and TKW, respectively, relative to the best single-trait and single-environment models.

**Table 2:** Model predictive abilities with different models for the three traits plant height, grain yield, and seed size (TKW) in the HSF population. Values in parenthesis are standard deviations. GBLUP, genomic best linear unbiased prediction; ridge-regression BLUP (rrBLUP).

Model	Trait	Env	Model type	Height	Yield	TKW
rrBLUP	Single	Single	Ridge-regression	−0.04 (0.10)	0.06 (0.12)	0.04 (0.10)
sommer	Single	Single	GBLUP	0.22 (0.06)	0.10 (0.06)	0.11 (0.09)
BRR	Single	Single	Bayesian	−0.07 (0.10)	0.04 (0.12)	0.04 (0.11)
MDe	Single	Multi	GBLUP	0.45 (0.04)	0.44 (0.03)	0.25 (0.03)
MTM	Multi	Single	Bayesian	0.11 (0.07)	0.16 (0.14)	0.13 (0.12)
MTM	Multi	Multi	Bayesian	0.21 (0.08)	0.19 (0.10)	0.17 (0.10)
BMTME	Multi	Multi	Bayesian	−0.01 (0.04)	0.34 (0.11)	0.11 (0.08)

**Fig 4:**
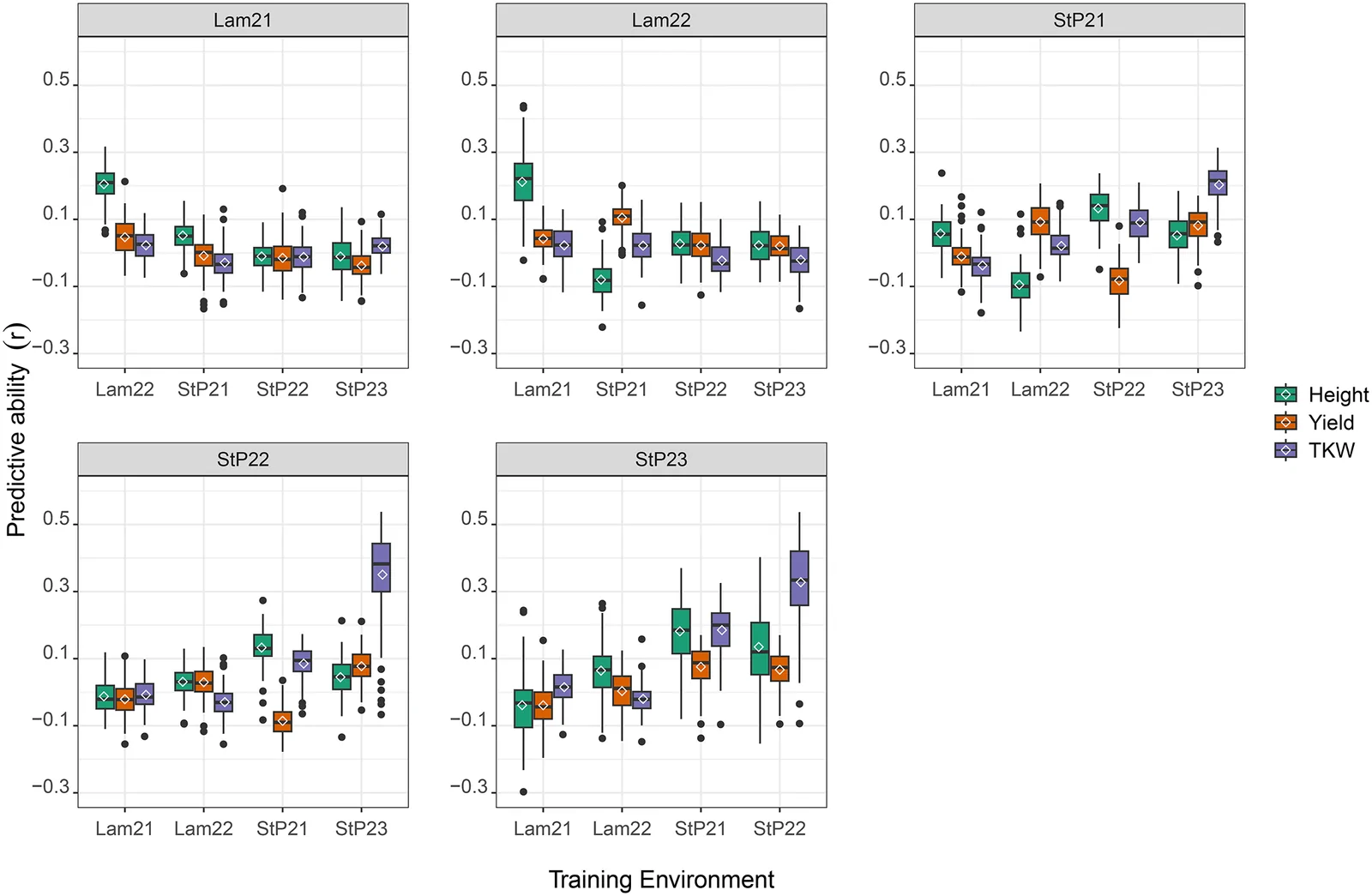
Cross-environment genomic predictions in the half-sib families for the traits plant height, grain yield, and seed size measured as thousand kernel weight (TKW). Each of the five environments, shown as facet labels in gray background at the top of each panel, was used to predict the remaining four environments with the multi-environment (MDe) model. White diamonds are mean values for each trait in each prediction scenario. Traits (x-axis) are grouped by training environments and predictive abilities (r) are shown on the y-axis.

We then assessed cross-environmental predictions by training the multi-environment model MDe. The model was trained with data from one environment and GEBVs for the remaining environments were predicted (Fig 4). The best cross-environment predictions were observed for seed size (TKW) when the 2022 St. Paul environment was predicted using data from 2023 St. Paul (predictive ability, *r* = 0.35), followed by prediction of the 2023 St. Paul environment using 2022 St. Paul data (*r* = 0.33). While no single environment was the overall best predictor of remaining environments, seed size was best predicted among the St. Paul environments with *r* ranging 0.09 to 0.35 and plant height was best predicted in the Lamberton environments (*r* = 0.21). Cross-environmental grain yield was predicted poorly as with *r* ranging −0.09 to 0.11.

Furthermore, the model was trained with data from two or more environments to predict GEBVs for the remaining single environments, i.e., the CV0 prediction scheme (S3 Table). Predictions obtained from combining two to three datasets were poorer relative to those obtained from single-environments (Fig 4). The highest predictions were observed for TKW (*r* = 0.16) in the 2023 St. Paul environment followed by plant height (*r* = 0.14) in the 2021 Lamberton environment when Lam22 and StP22 were combined. Grain yield was worse-predicted with *r* values ranging from −0.05 to 0.08. Combination of all three St. Paul datasets poorly predicted trait performance in Lamberton, and combination of both Lamberton datasets did not predict StP well. This was expected given the stark contrast between the two locations for trait expression, as shown in Fig 3D. No particular combination exceeded the prediction values observed from models trained with single-environment datasets.

Estimates of expected genetic gain, calculated using the predictive abilities of models trained with single-environment datasets, for the three traits ranged −0.7 to 1.8, with the negative value indicating a reduction in plant height (Table 3). In terms of realized gain, the largest potential improvement was observed for grain yield where an increase of approximately 19.97 kg ha^-1^ per breeding cycle could be obtained. Given that the lowest model predictive ability was for seed size (TKW, Table 2), the smallest gain was observed for this trait with ΔG value of nearly zero and size increase of < 0.02 g per breeding cycle.

**Table 3:** Expected genetic gain in the IWG half-sib families from the UMN breeding program. Genetic gain was estimated using predictive abilities from multivariate genomic prediction models. Here, σ^2^_A_ is additive genetic variance; σ_P_ is phenotypic distribution standard deviation; i is selection intensity and a negative value indicates reduction in trait mean; *r* is the highest predictive ability obtained from multivariate models; ΔG is the expected genetic gain, and ΔG_pheno_ is the realized gain, i.e., expected change in phenotype values based on ΔG.

Trait	σ^2^_A_	σ_P_	i	r	ΔG	ΔG_pheno_
Plant height (cm)	44.77	12.00	−0.22	0.45	−0.66	−3.29
Grain yield (kg ha^-1^)	24.57	15.07	0.82	0.44	1.79	19.97
TKW (g)	0.39	1.43	0.24	0.25	0.04	0.02

## Discussion

The 208 HSFs evaluated in this study represented the overall genetic diversity of the UMN’s Cycle 4 breeding population [25]. Despite all germplasm being sampled from a single population, we observed little relatedness among the maternal genotypes of the HSFs (Fig 1). Perhaps owing to a weaker relatedness among the maternal genotypes, i.e., more diverse genotypes, a large number (31,640) of high-quality genome-wide SNPs were discovered and had less than 10% missing allelic information.

Trait response among the HSFs varied remarkably across the two locations and three years (Fig 2, Fig 3), with more year-to-year variations in trait responses in St. Paul compared to Lamberton. Yet, the two locations were more similar in their expression of the traits plant height and seed size irrespective of the evaluation year. In other words, genotype by location interaction effects dominate expression of height and seed size but yield response was more affected by genotype by year effect. Both locations chosen for this experiment resemble the environments where most of the IWG/Kernza cultivation fields are currently located in Minnesota, USA. Also, the differences in trait expression across the environments had a noticeable impact on genetic estimates of trait heritabilities. As reported in our former study for the same population, the broad sense trait heritabilities (*H*^*2*^) were 0.36 for grain yield, 0.51 for plant height, and 0.44 for TKW [25]. While the *H*^*2*^ estimates for yield and height remained within previously reported estimates observed in our past few breeding populations [16,17,26], the estimates obtained for TKW were surprisingly low. To elucidate: strong *H*^*2*^ estimates have been observed in the past for TKW within the UMN IWG breeding program, ranging 0.69–0.85 [15–17,26]. We propose two plausible explanations behind these observations: 1) the HSFs represent different population composition relative to spaced-planted breeding populations that had predominantly been used in the past experiments and breeding trials, and 2) large changes in environmental parameters, namely temperature and rainfall, during the years this experiment was conducted relative to previous years. For example, both Lamberton and St. Paul experienced less rainfall and higher temperatures during the months of May-August in all three years compared to the same months between 2000 and 2020 (S4 Table). The hotter and drier conditions experienced by the HSFs during their field evaluations in the two MN locations led to a decrease in the mean seed size across all trial years and locations (TKW = 6.85 g) relative to previous breeding cycles (TKW range of 6.87 g – 8.33 g, S5 Table) that were evaluated in higher rainfall conditions. This in turn, reduced the trait variance in the HSFs (S5 Table) and therefore contributed to reduced *H*^*2*^ observed in our analysis. As breeding programs continue to evaluate additional HSFs in multi-year experiments in diverse locations, further clarity regarding the inter-relationship among the genetic and non-genetic factors may be obtained. Beginning with the 2025 growing season, we have replaced our Lamberton evaluation location with Becker, a location that has capabilities to irrigate, if necessary, to better ensure greater trait expression.

The model predictions observed in this population of HSFs were lower than the values reported in other IWG breeding populations. Seed size (TKW) had the lowest predictions compared to other traits, which is likely because of the lower heritability observed for TKW in this study. This was a surprising observation as our prior reports have found TKW to typically have higher heritability estimates and stronger model predictions [16,17]. Studies conducted in multiple crop species have shown that model predictive ability is affected by trait heritability, with lower predictions observed when heritability estimates are low [11,36]. However, it must be noted that past studies conducted by the UMN program and others have used spaced-planted nurseries composed of individual genotypes to assess GS models. For example, predictions in the UMN breeding populations by univariate and multivariate models ranged 0.23–0.52 for grain yield, 0.56–0.62 for plant height, and 0.55–0.78 for TKW [16,17,26]. Likewise, in other IWG breeding programs, these values have ranged from 0.02–0.58 for yield, 0.18–0.56 for height, and 0.35–0.67 for TKW [49,50]. In addition to the inherent difference in population type, it should also be noted that 1) the spaced-planted populations were evaluated in locations and years different from these HSFs, eliciting differences in trait expression that affect predictions, and 2) genetic variance in HSFs is 1/4th that of the spaced-planted nurseries [19]. Collectively, these factors are expected to influence the predictive ability of GS models [51,52]. Yet another possible explanation behind the lower predictions is that the training set size used in this study is smaller than those that have been used in past IWG research. Smaller training sets, when unoptimized for target breeding population and traits of interests, have been known to result in lower model accuracies [53,54].

Despite obtaining lower predictions compared to past IWG reports, our study reconfirmed that inclusion of genotype by environment interaction effects improved trait predictions. Such observations have been made by several other crop species where multivariate models are more effective than univariate models [55–57]. The multivariate prediction models are able to extract more reliable information on phenotypic performance of the same population from multiple diverse environments [42]. We had observed good cross-environmental, i.e., cross-location and cross-year, predictions in the UMN-C3 population where the first year trait data collected in St. Paul was able to predict remaining environments with moderate to good predictions with model predictive abilities ranging from 0.31–0.37 for grain yield and 0.57–0.58 for TKW; plant height was not predicted [18]. We therefore conducted cross-environmental predictions in these HSFs to evaluate if trait performance from one trial or environment could be used to predict values in other environments. This was of interest to determine if 1) the breeding program can move towards a less resource-intensive operation by reducing the experimental footprint over multiple years and locations, and 2) good predictions can be used as proxies for untested environments in contingencies where a trial might be lost from poor maintenance or natural disasters. We observed that the St. Paul environments were reasonably adequate in predicting seed size in other St. Paul environments but not other traits. Yet this was not the case with the Lamberton environments where all trait predictions were poor. Combination of all three St. Paul datasets poorly predicted trait performance in Lamberton, and combination of both Lamberton datasets did not predict plant performance in St. Paul well. This was expected given the stark contrast between the locations for trait expression, as shown in Fig 3D. No particular combination exceeded the prediction values observed from models trained with single-environment datasets. Similarly, poor observations were made when data collected in one year at both locations were used to predict trait values in all remaining environments (S3l Table). The clear lack of strong cross-location and cross-annum predictions suggests that maintaining a selection nursery in a second location over multiple growing seasons is important, considering that inclusion of multi-location trait data boosts trait predictions. Nonetheless, the ability to predict some traits across independent years within one location could be expected to reduce some resource and labor inputs for this location and allocate resources elsewhere in the breeding program.

Since genomic prediction models were first implemented in plant breeding programs, it has been well established that models with higher predictive abilities can greatly improve the accuracy of plant selections [58]. This is true also for quantitative and complex traits such as grain yield which is thought to be influenced by several genetic factors and environmental variations [59]. In our study, we found that grain yield and plant height, complex traits in IWG, had good predictions yet seed size did not. As expected, this variability in trait prediction affects the estimated genetic gain dependent on model predictive abilities. The higher predictions reported by multivariate models are promising and should lead to meaningful improvements in increasing grain yield and reducing plant height. Of note: the largest estimated gains were observed for grain yield: 1.79 units or 19.97 kg ha^-1^ increase per breeding cycle on average. This is an encouraging metric as IWG yield is not only lower compared to mainstream summer and winter-annual cereal crops, the seed yield also begins to decline in year 2 of crop cultivation [4,24]. The estimated gain for plant height was −0.66 units or a reduction of 3.29 cm per breeding cycle. This is a desirable outcome as shorter plants, in general, could be expected to experience less lodging as the plants mature thereby improving harvest efficiency for both biomass and grain yield. The sub-optimal realized gain for seed size, while not ideal, could see an improvement as additional HSFs are evaluated in additional years and locations within and outside the UMN program. We anticipate that the adoption of row-based experiments by breeding programs for genetic evaluation and recurrent selection schemes could assist in improving grain yield while potentially addressing yield decline in this crop. Paired with multivariate genomic prediction models that offer robust and more accurate predictions, individuals with promise of high yield and less yield decline can be selected to create newer breeding populations.

## Conclusions

In this study, we assessed genomic prediction models and estimated genetic gain in large half-sib IWG families evaluated over two locations and three years for grain yield, seed size, and plant height. We found that it is possible to obtain good predictions when genomic prediction models are used to predict trait performance in HSFs, yet not all traits are predicted with the same robustness. Multivariate models that integrated multi-dimensional relationships were noticeably better than univariate models in trait predictions. In a previous study that reported results from a genome-wide association scan in the same population as the one discussed here, we identified germplasm with no yield decline in IWG. The best prediction models discussed in this study could therefore be used to select genotypes that have little or no yield decline. Making crosses with genotypes to develop progeny that do not exhibit yield declines with stand age could greatly contribute to overcoming this important barrier to large-scale perennial grain production. As more IWG breeding programs routinely adopt HSFs in their experiments and selection regime, we anticipate that these models can be further trained to improve prediction accuracies and improve breeding populations.

## Supporting information

S1 Table Trait data of mother plants from the UMN Cycle 4 population from which the half-sib families were obtained. (DOCX)

S2 Table Trait data (BLUEs) for the half-sib families in Lamberton, MN and St. Paul, MN in 2021, 2022, and 2023. (DOCX)

S3 Table Cross-environment genomic predictions in the half-sib families for the traits plant height, grain yield, and seed size measured as thousand kernel weight (TKW). Multiple environment predictions were made with the multi-environment (MDe) model with training populations shows in rows and predicted populations in columns.
(DOCX)

S4 Table Weather data of Lamberton, MN and St. Paul, MN between 2020 and 2023. Information obtained from https://www.weather.gov; accessed 03-17-25.
(DOCX)

S5 Table Trait mean and variance in UMN IWG breeding cycles C1-C5 and the half-sib families. Note that C1-C5 yield are per plant basis while that for HSFs and checks is on per hectare basis.
(DOCX)
