# Supplementary material for: Assessment of genomic prediction and genetic gain in multi‑population half-sib families in the perennial grass crop intermediate wheatgrass
Source: PLoS One. 2026 Sep 2;21(9):e0357452. doi: 10.1371/journal.pone.0357452 (PMC13537581; doi:10.1371/journal.pone.0357452)
Supplement: Supplementary file 1 [file pone.0357452.s001.docx]

**S1 Table:** Trait data of mother plants from the UMN Cycle 4 population from which the half-sib families were obtained.

| **C4_Mother** | **HSF_ID** | **Location** | **Year** | **Shatter_resistance** | **Threshability** | **Yield_g** | **TKW_g** | **Height_cm** |
| --- | --- | --- | --- | --- | --- | --- | --- | --- |
| C418S0213 | C4HSF001 | StP | 2019 | 5 | 8.00 | 32.47 | 8.74 | 127.5 |
| C418S0228 | C4HSF002 | StP | 2019 | 6 | 6.00 | 41.92 | 11.80 | 125 |
| C418S0252 | C4HSF003 | StP | 2019 | 0 | 9.00 | 30.53 | 8.59 | 112.5 |
| C418S0263 | C4HSF004 | StP | 2019 | 2 | 1.00 | 34.11 | 9.81 | 120 |
| C418S0284 | C4HSF005 | StP | 2019 | 2 | 6.00 | 23.00 | 8.21 | 102.5 |
| C418S0286 | C4HSF006 | StP | 2019 | 2 | 2.00 | 33.41 | 8.63 | 125 |
| C418S0425 | C4HSF007 | StP | 2019 | 0 | 6.00 | 4.26 | 9.39 | 125 |
| C418S0469 | C4HSF008 | StP | 2019 | 3 | 8.00 | 26.60 | 8.35 | 135 |
| C418S0499 | C4HSF009 | StP | 2019 | 2 | 7.00 | 6.35 | 7.43 | 112.5 |
| C418S0565 | C4HSF010 | StP | 2019 | 5 | 1.00 | 39.36 | 7.21 | 130 |
| C418S0619 | C4HSF011 | StP | 2019 | 0 | 9.00 | NA | 9.68 | 127.5 |
| C418S0003 | C4HSF012 | StP | 2019 | 1 | 6.00 | 53.89 | 10.67 | 102.5 |
| C418S0004 | C4HSF013 | StP | 2019 | 6 | 4.00 | 46.31 | 14.09 | 112.5 |
| C418S0006 | C4HSF014 | StP | 2019 | 2 | 7.00 | 38.65 | 11.33 | 102.5 |
| C418S0007 | C4HSF015 | StP | 2019 | 0 | 8.00 | 50.23 | 10.04 | 135 |
| C418S0008 | C4HSF016 | StP | 2019 | 1 | 8.00 | 31.66 | 8.68 | 130 |
| C418S0009 | C4HSF017 | StP | 2019 | 5 | 4.00 | 39.63 | 11.02 | 125 |
| C418S0010 | C4HSF018 | StP | 2019 | 0 | 7.00 | 37.16 | 8.65 | 132.5 |
| C418S0014 | C4HSF019 | StP | 2019 | 0 | 1.00 | 42.37 | 10.65 | 135 |
| C418S0015 | C4HSF020 | StP | 2019 | 0 | 2.00 | 62.07 | 9.64 | 120 |
| C418S0017 | C4HSF021 | StP | 2019 | 3 | 1.00 | 35.56 | 9.82 | 132.5 |
| C418S0020 | C4HSF022 | StP | 2019 | 0 | 6.00 | 58.64 | 10.36 | 127.5 |
| C418S0021 | C4HSF023 | StP | 2019 | 1 | 2.00 | 36.66 | 7.57 | 142.5 |
| C418S0022 | C4HSF024 | StP | 2019 | 9 | 1.00 | 61.53 | 9.41 | 140 |
| C418S0023 | C4HSF025 | StP | 2019 | 2 | 3.00 | 53.59 | 10.92 | 147.5 |
| C418S0024 | C4HSF026 | StP | 2019 | 1 | 9.00 | 60.09 | 10.30 | 127.5 |
| C418S0025 | C4HSF027 | StP | 2019 | 7 | 3.00 | 47.85 | 11.08 | 142.5 |
| C418S0026 | C4HSF028 | StP | 2019 | 6 | 7.00 | 73.04 | 9.98 | 135 |
| C418S0028 | C4HSF029 | StP | 2019 | 5 | 6.00 | 48.22 | 10.45 | 112.5 |
| C418S0031 | C4HSF030 | StP | 2019 | 6 | 4.00 | 43.85 | 13.30 | 125 |
| C418S0032 | C4HSF031 | StP | 2019 | 7 | 3.00 | 114.51 | 10.49 | 127.5 |
| C418S0034 | C4HSF032 | StP | 2019 | 1 | 6.00 | 71.84 | 10.78 | 130 |
| C418S0035 | C4HSF033 | StP | 2019 | 5 | 1.00 | 52.75 | 15.26 | 127.5 |
| C418S0051 | C4HSF034 | StP | 2019 | 3 | 7.00 | 47.49 | 10.19 | 117.5 |
| C418S0054 | C4HSF035 | StP | 2019 | 6 | 6.00 | 45.05 | 9.95 | 125 |
| C418S0056 | C4HSF036 | StP | 2019 | 3 | 7.00 | 32.14 | 9.33 | 120 |
| C418S0061 | C4HSF037 | StP | 2019 | 0 | 4.00 | 60.14 | 6.58 | 125 |
| C418S0062 | C4HSF038 | StP | 2019 | 6 | 3.00 | 80.21 | 9.50 | 127.5 |
| C418S0063 | C4HSF039 | StP | 2019 | 2 | 9.00 | 75.72 | 11.83 | 120 |
| C418S0068 | C4HSF040 | StP | 2019 | 0 | 6.00 | 75.79 | 10.16 | 125 |
| C418S0079 | C4HSF041 | StP | 2019 | 6 | 4.00 | 44.59 | 10.17 | 117.5 |
| C418S0084 | C4HSF042 | StP | 2019 | 2 | 8.00 | 49.47 | 9.65 | 140 |
| C418S0086 | C4HSF043 | StP | 2019 | 1 | 3.00 | 39.85 | 8.70 | 110 |
| C418S0087 | C4HSF044 | StP | 2019 | 5 | 8.00 | 44.68 | 11.47 | 137.5 |
| C418S0089 | C4HSF045 | StP | 2019 | 0 | 9.00 | 38.95 | 10.67 | 130 |
| C418S0092 | C4HSF046 | StP | 2019 | 1 | 1.00 | 35.57 | 7.23 | 95 |
| C418S0093 | C4HSF047 | StP | 2019 | 2 | 3.00 | 41.93 | 9.65 | 133.5 |
| C418S0097 | C4HSF049 | StP | 2019 | 2 | 6.00 | 42.94 | 9.08 | 135 |
| C418S0098 | C4HSF050 | StP | 2019 | 2 | 6.00 | 45.20 | 10.47 | 145 |
| C418S0100 | C4HSF051 | StP | 2019 | 6 | 5.00 | 58.04 | 10.08 | 150 |
| C418S0105 | C4HSF052 | StP | 2019 | 5 | 7.00 | 70.15 | 10.83 | 135 |
| C418S0118 | C4HSF054 | StP | 2019 | 0 | 6.00 | 74.08 | 10.22 | 130 |
| C418S0119 | C4HSF055 | StP | 2019 | 3 | 5.00 | 56.89 | 9.43 | 150 |
| C418S0120 | C4HSF056 | StP | 2019 | 2 | 3.00 | 48.61 | 10.54 | 135 |
| C418S0121 | C4HSF057 | StP | 2019 | 0 | 9.00 | 40.97 | 13.49 | 140 |
| C418S0122 | C4HSF058 | StP | 2019 | 0 | 9.00 | 84.79 | 9.67 | 135 |
| C418S0124 | C4HSF059 | StP | 2019 | 0 | 2.00 | 45.59 | 7.86 | 137.5 |
| C418S0125 | C4HSF060 | StP | 2019 | 5 | 7.00 | 48.40 | 8.75 | 127.5 |
| C418S0126 | C4HSF061 | StP | 2019 | 4 | 6.00 | 63.73 | 10.85 | 130 |
| C418S0128 | C4HSF062 | StP | 2019 | 4 | 0.00 | 51.64 | 11.97 | 140 |
| C418S0129 | C4HSF063 | StP | 2019 | 9 | 0.00 | 66.95 | 10.75 | 132.5 |
| C418S0131 | C4HSF064 | StP | 2019 | 1 | 1.00 | 40.17 | 9.35 | 142.5 |
| C418S0133 | C4HSF065 | StP | 2019 | 2 | 7.00 | 41.52 | 9.26 | 127.5 |
| C418S0134 | C4HSF066 | StP | 2019 | 0 | 5.00 | 47.86 | 10.24 | 137.5 |
| C418S0135 | C4HSF067 | StP | 2019 | 3 | 4.00 | 35.83 | 9.60 | 117.5 |
| C418S0137 | C4HSF068 | StP | 2019 | 5 | 0.00 | 43.85 | 11.11 | 120 |
| C418S0138 | C4HSF069 | StP | 2019 | 5 | 0.00 | 61.53 | 11.05 | 135 |
| C418S0139 | C4HSF070 | StP | 2019 | 5 | 4.00 | 38.44 | 9.63 | 127.5 |
| C418S0140 | C4HSF071 | StP | 2019 | 7 | 3.00 | 43.27 | 9.47 | 130 |
| C418S0143 | C4HSF073 | StP | 2019 | 5 | 5.00 | 52.34 | 9.73 | 132.5 |
| C418S0145 | C4HSF074 | StP | 2019 | 5 | 7.00 | 68.69 | 8.94 | 127.5 |
| C418S0149 | C4HSF076 | StP | 2019 | 3 | 6.00 | 32.80 | 8.26 | 135 |
| C418S0150 | C4HSF077 | StP | 2019 | 0 | 9.00 | 30.99 | 7.68 | 102.5 |
| C418S0151 | C4HSF078 | StP | 2019 | 2 | 7.00 | 52.41 | 8.57 | 130 |
| C418S0152 | C4HSF079 | StP | 2019 | 2 | 7.00 | 33.65 | 7.43 | 112.5 |
| C418S0153 | C4HSF080 | StP | 2019 | 3 | 4.00 | 31.03 | 7.95 | 120 |
| C418S0154 | C4HSF081 | StP | 2019 | 5 | 5.00 | 60.16 | 11.12 | 117.5 |
| C418S0156 | C4HSF082 | StP | 2019 | 2 | 1.00 | 30.52 | 10.53 | 125 |
| C418S0157 | C4HSF083 | StP | 2019 | 2 | 3.00 | 34.32 | 9.75 | 95 |
| C418S0161 | C4HSF084 | StP | 2019 | 2 | 2.00 | 75.20 | 10.59 | 115 |
| C418S0163 | C4HSF085 | StP | 2019 | 6 | 6.00 | 32.68 | 8.68 | 125 |
| C418S0164 | C4HSF086 | StP | 2019 | 3 | 7.00 | 46.50 | 7.86 | 140 |
| C418S0165 | C4HSF087 | StP | 2019 | 6 | 7.00 | 30.58 | 9.39 | 125 |
| C418S0166 | C4HSF088 | StP | 2019 | 0 | 9.00 | 35.10 | 9.17 | 135 |
| C418S0167 | C4HSF089 | StP | 2019 | 5 | 2.00 | 38.68 | 9.56 | 120 |
| C418S0168 | C4HSF090 | StP | 2019 | 6 | 7.00 | 53.70 | 11.22 | 150 |
| C418S0169 | C4HSF091 | StP | 2019 | 7 | 4.00 | 65.28 | 10.18 | 125 |
| C418S0171 | C4HSF092 | StP | 2019 | 6 | 6.00 | 60.44 | 8.41 | 132.5 |
| C418S0172 | C4HSF093 | StP | 2019 | 4 | 6.00 | 33.11 | 10.07 | 132.5 |
| C418S0177 | C4HSF094 | StP | 2019 | 5 | 0.00 | 42.60 | 9.87 | 150 |
| C418S0178 | C4HSF095 | StP | 2019 | 5 | 0.00 | 31.25 | 7.29 | 137.5 |
| C418S0180 | C4HSF096 | StP | 2019 | 7 | 0.00 | 54.22 | 11.66 | 132.5 |
| C418S0193 | C4HSF097 | StP | 2019 | 7 | 8.00 | 34.86 | 11.59 | 125 |
| C418S0196 | C4HSF098 | StP | 2019 | 5 | 7.00 | 37.76 | 9.14 | 152.5 |
| C418S0197 | C4HSF099 | StP | 2019 | 6 | 0.00 | 32.25 | 9.43 | 115 |
| C418S0199 | C4HSF100 | StP | 2019 | 2 | 5.00 | 61.11 | 11.31 | 135 |
| C418S0201 | C4HSF101 | StP | 2019 | 1 | 8.00 | 34.35 | 9.57 | 135 |
| C418S0202 | C4HSF102 | StP | 2019 | 0 | 7.00 | 57.48 | 9.25 | 132.5 |
| C418S0206 | C4HSF103 | StP | 2019 | 1 | 8.00 | 46.45 | 12.02 | 122.5 |
| C418S0207 | C4HSF104 | StP | 2019 | 0 | 9.00 | 38.13 | 10.86 | 115 |
| C418S0208 | C4HSF105 | StP | 2019 | 1 | 9.00 | 33.62 | 9.06 | 115 |
| C418S0209 | C4HSF106 | StP | 2019 | 2 | 6.00 | 46.09 | 11.51 | 127.5 |
| C418S0210 | C4HSF107 | StP | 2019 | 2 | 9.00 | 79.66 | 11.10 | 140 |
| C418S0215 | C4HSF108 | StP | 2019 | 3 | 4.00 | 72.31 | 10.03 | 127.5 |
| C418S0217 | C4HSF109 | StP | 2019 | 0 | 8.00 | 42.57 | 8.62 | 120 |
| C418S0219 | C4HSF110 | StP | 2019 | 2 | 4.00 | 69.87 | 8.79 | 120 |
| C418S0220 | C4HSF111 | StP | 2019 | 0 | 3.00 | 45.10 | 7.77 | 117.5 |
| C418S0225 | C4HSF112 | StP | 2019 | 2 | 7.00 | 38.93 | 7.83 | 102.5 |
| C418S0226 | C4HSF113 | StP | 2019 | 0 | 5.00 | 57.74 | 10.67 | 140 |
| C418S0230 | C4HSF114 | StP | 2019 | 5 | 4.00 | 30.79 | 8.79 | 122.5 |
| C418S0232 | C4HSF115 | StP | 2019 | 0 | 8.00 | 37.52 | 9.81 | 117.5 |
| C418S0233 | C4HSF116 | StP | 2019 | 0 | 6.00 | 62.70 | 10.36 | 132.5 |
| C418S0234 | C4HSF117 | StP | 2019 | 7 | 0.00 | 33.16 | 10.27 | 142.5 |
| C418S0235 | C4HSF118 | StP | 2019 | 9 | 2.00 | 46.46 | 10.63 | 150 |
| C418S0236 | C4HSF119 | StP | 2019 | 3 | 3.00 | 83.10 | 9.36 | 132.5 |
| C418S0237 | C4HSF120 | StP | 2019 | 5 | 4.00 | 50.09 | 8.91 | 125 |
| C418S0240 | C4HSF121 | StP | 2019 | 7 | 1.00 | 33.86 | 11.43 | 150 |
| C418S0242 | C4HSF122 | StP | 2019 | 6 | 1.00 | 37.63 | 7.75 | 142.5 |
| C418S0243 | C4HSF123 | StP | 2019 | 5 | 3.00 | 72.12 | 8.27 | 135 |
| C418S0244 | C4HSF124 | StP | 2019 | 0 | 3.00 | 42.70 | 7.79 | 137.5 |
| C418S0245 | C4HSF125 | StP | 2019 | 0 | 9.00 | 31.94 | 10.73 | 127.5 |
| C418S0253 | C4HSF126 | StP | 2019 | 8 | 2.00 | 44.96 | 9.45 | 127.5 |
| C418S0254 | C4HSF127 | StP | 2019 | 7 | 2.00 | 37.92 | 7.49 | 117.5 |
| C418S0259 | C4HSF128 | StP | 2019 | 3 | 7.00 | 39.75 | 12.62 | 140 |
| C418S0260 | C4HSF129 | StP | 2019 | 0 | 8.00 | 35.58 | 9.82 | 127.5 |
| C418S0264 | C4HSF130 | StP | 2019 | 7 | 5.00 | 36.79 | 11.63 | 115 |
| C418S0267 | C4HSF131 | StP | 2019 | 2 | 8.00 | 35.58 | 9.10 | 130 |
| C418S0268 | C4HSF132 | StP | 2019 | 7 | 4.00 | 50.31 | 11.76 | 115 |
| C418S0269 | C4HSF133 | StP | 2019 | 2 | 9.00 | 79.04 | 10.04 | 132.5 |
| C418S0270 | C4HSF134 | StP | 2019 | 6 | 7.00 | 77.26 | 10.09 | 135 |
| C418S0273 | C4HSF135 | StP | 2019 | 1 | 4.00 | 65.05 | 10.03 | 135 |
| C418S0274 | C4HSF136 | StP | 2019 | 3 | 8.00 | 32.38 | 10.81 | 127.5 |
| C418S0276 | C4HSF137 | StP | 2019 | 1 | 8.00 | 38.42 | 7.58 | 130 |
| C418S0278 | C4HSF138 | StP | 2019 | 4 | 6.00 | 47.67 | 8.52 | 135 |
| C418S0280 | C4HSF139 | StP | 2019 | 1 | 0.00 | 78.82 | 8.46 | 125 |
| C418S0281 | C4HSF140 | StP | 2019 | 2 | 1.00 | 61.41 | 6.82 | 130 |
| C418S0282 | C4HSF141 | StP | 2019 | 6 | 5.00 | 39.57 | 8.71 | 110 |
| C418S0283 | C4HSF142 | StP | 2019 | 3 | 5.00 | 37.63 | 8.59 | 117.5 |
| C418S0285 | C4HSF143 | StP | 2019 | 2 | 1.00 | 43.26 | 7.60 | 105 |
| C418S0289 | C4HSF144 | StP | 2019 | 7 | 6.00 | 39.45 | 10.08 | 127.5 |
| C418S0291 | C4HSF145 | StP | 2019 | 5 | 7.00 | 52.42 | 10.55 | 115 |
| C418S0298 | C4HSF146 | StP | 2019 | 3 | 2.00 | 50.56 | 8.15 | 137.5 |
| C418S0301 | C4HSF147 | StP | 2019 | 2 | 5.00 | 43.98 | 8.00 | 132.5 |
| C418S0302 | C4HSF148 | StP | 2019 | 6 | 4.00 | 30.36 | 11.26 | 135 |
| C418S0303 | C4HSF149 | StP | 2019 | 2 | 1.00 | 56.96 | 10.63 | 137.5 |
| C418S0304 | C4HSF150 | StP | 2019 | 2 | 2.00 | 32.40 | 10.49 | 120 |
| C418S0305 | C4HSF151 | StP | 2019 | 6 | 1.00 | 38.84 | 11.52 | 130 |
| C418S0308 | C4HSF152 | StP | 2019 | 0 | 4.00 | 36.97 | 8.49 | 112.5 |
| C418S0309 | C4HSF153 | StP | 2019 | 3 | 8.00 | 33.82 | 8.70 | 102.5 |
| C418S0315 | C4HSF154 | StP | 2019 | 0 | 8.00 | 35.67 | 8.17 | 110 |
| C418S0321 | C4HSF155 | StP | 2019 | 0 | 9.00 | 38.79 | 9.09 | 122.5 |
| C418S0325 | C4HSF156 | StP | 2019 | 2 | 8.00 | 51.34 | 9.09 | 122.5 |
| C418S0327 | C4HSF157 | StP | 2019 | 2 | 6.00 | 35.62 | 10.61 | 125 |
| C418S0330 | C4HSF158 | StP | 2019 | 7 | 4.00 | 54.92 | 9.65 | 137.5 |
| C418S0336 | C4HSF159 | StP | 2019 | 2 | 9.00 | 41.22 | 9.41 | 137.5 |
| C418S0337 | C4HSF160 | StP | 2019 | 7 | 7.00 | 30.35 | 10.41 | 122.5 |
| C418S0340 | C4HSF161 | StP | 2019 | 3 | 6.00 | 33.88 | 8.46 | 152.5 |
| C418S0343 | C4HSF162 | StP | 2019 | 2 | 9.00 | 52.05 | 11.31 | 120 |
| C418S0344 | C4HSF163 | StP | 2019 | 7 | 4.00 | 45.02 | 11.34 | 120 |
| C418S0350 | C4HSF164 | StP | 2019 | 5 | 5.00 | 35.98 | 10.80 | 112.5 |
| C418S0351 | C4HSF165 | StP | 2019 | 0 | 8.00 | 31.16 | 9.45 | 127.5 |
| C418S0352 | C4HSF166 | StP | 2019 | 0 | 9.00 | 35.44 | 8.37 | 125 |
| C418S0353 | C4HSF167 | StP | 2019 | 6 | 5.00 | 53.85 | 10.06 | 160 |
| C418S0355 | C4HSF168 | StP | 2019 | 1 | 6.00 | 38.91 | 8.61 | 147.5 |
| C418S0356 | C4HSF169 | StP | 2019 | 1 | 8.00 | 41.87 | 9.41 | 157.5 |
| C418S0357 | C4HSF170 | StP | 2019 | 0 | 3.00 | 30.54 | 11.35 | 140 |
| C418S0358 | C4HSF171 | StP | 2019 | 0 | 7.00 | 46.99 | 7.58 | 140 |
| C418S0360 | C4HSF172 | StP | 2019 | 7 | 6.00 | 71.72 | 11.57 | 132.5 |
| C418S0362 | C4HSF173 | StP | 2019 | 5 | 8.00 | 40.93 | 11.08 | 145 |
| C418S0364 | C4HSF174 | StP | 2019 | 7 | 4.00 | 49.94 | 8.57 | 127.5 |
| C418S0365 | C4HSF175 | StP | 2019 | 6 | 7.00 | 40.77 | 8.63 | 130 |
| C418S0367 | C4HSF176 | StP | 2019 | 2 | 7.00 | 41.56 | 9.11 | 140 |
| C418S0368 | C4HSF177 | StP | 2019 | 7 | 8.00 | 52.22 | 10.05 | 132.5 |
| C418S0369 | C4HSF178 | StP | 2019 | 6 | 4.00 | 37.93 | 8.81 | 140 |
| C418S0382 | C4HSF179 | StP | 2019 | 0 | 9.00 | 43.69 | 6.81 | 122.5 |
| C418S0385 | C4HSF180 | StP | 2019 | 0 | 7.00 | 52.78 | 8.06 | 115 |
| C418S0386 | C4HSF181 | StP | 2019 | 2 | 4.00 | 31.93 | 8.63 | 122.5 |
| C418S0390 | C4HSF182 | StP | 2019 | 8 | 1.00 | 67.93 | 13.84 | 130 |
| C418S0391 | C4HSF183 | StP | 2019 | 2 | 4.00 | 32.03 | 11.05 | 137.5 |
| C418S0392 | C4HSF184 | StP | 2019 | 1 | 7.00 | 45.42 | 8.95 | 132.5 |
| C418S0394 | C4HSF185 | StP | 2019 | 1 | 9.00 | 53.24 | 10.70 | 147.5 |
| C418S0396 | C4HSF186 | StP | 2019 | 0 | 2.00 | 31.20 | 9.95 | 132.5 |
| C418S0399 | C4HSF187 | StP | 2019 | 7 | 3.00 | 38.54 | 9.36 | 112.5 |
| C418S0401 | C4HSF188 | StP | 2019 | 6 | 3.00 | 39.69 | 13.47 | 140 |
| C418S0406 | C4HSF189 | StP | 2019 | 6 | 8.00 | 33.99 | 8.92 | 132.5 |
| C418S0410 | C4HSF190 | StP | 2019 | 0 | 5.00 | 40.17 | 8.79 | 133.5 |
| C418S0411 | C4HSF191 | StP | 2019 | 7 | 7.00 | 48.78 | 11.12 | 145 |
| C418S0412 | C4HSF192 | StP | 2019 | 2 | 4.00 | 48.98 | 8.78 | 145 |
| C418S0413 | C4HSF193 | StP | 2019 | 7 | 6.00 | 36.84 | 9.40 | 142.5 |
| C418S0414 | C4HSF194 | StP | 2019 | 0 | 6.00 | 32.08 | 8.70 | 162.5 |
| C418S0420 | C4HSF195 | StP | 2019 | 4 | 6.00 | 42.61 | 9.07 | 115 |
| C418S0421 | C4HSF196 | StP | 2019 | 3 | 2.00 | 39.29 | 10.41 | 132.5 |
| C418S0422 | C4HSF197 | StP | 2019 | 4 | 7.00 | 37.28 | 9.10 | 122.5 |
| C418S0423 | C4HSF198 | StP | 2019 | 1 | 5.00 | 43.96 | 12.08 | 125 |
| C418S0426 | C4HSF199 | StP | 2019 | 7 | 2.00 | 58.54 | 10.24 | 130 |
| C418S0427 | C4HSF200 | StP | 2019 | 7 | 8.00 | 35.33 | 9.93 | 140 |
| C418S0428 | C4HSF201 | StP | 2019 | 4 | 7.00 | 70.81 | 9.23 | 145 |
| C418S0429 | C4HSF202 | StP | 2019 | 0 | 8.00 | 72.64 | 8.09 | 127.5 |
| C418S0430 | C4HSF203 | StP | 2019 | 0 | 9.00 | 66.46 | 9.26 | 147.5 |
| C418S0455 | C4HSF204 | StP | 2019 | 0 | 8.00 | 63.24 | 11.05 | 142.5 |
| C418S0458 | C4HSF205 | StP | 2019 | 2 | 4.00 | 62.25 | 9.87 | 125 |
| C418S0463 | C4HSF206 | StP | 2019 | 5 | 1.00 | 71.58 | 6.26 | 112.5 |
| C418S0489 | C4HSF211 | StP | 2019 | 7 | 6.00 | 65.44 | 10.76 | 142.5 |
| C418S0491 | C4HSF212 | StP | 2019 | 0 | 9.00 | 64.79 | 9.85 | 145 |
| C418S0492 | C4HSF213 | StP | 2019 | 2 | 7.00 | 71.23 | 9.12 | 127.5 |
| C418S0497 | C4HSF217 | StP | 2019 | 0 | 6.00 | 63.19 | 8.04 | 147.5 |
| C418S0572 | C4HSF223 | StP | 2019 | 5 | 4.00 | 61.62 | 9.91 | 140 |
