# Supplementary material for: Assessment of genomic prediction and genetic gain in multi‑population half-sib families in the perennial grass crop intermediate wheatgrass
Source: PLoS One. 2026 Sep 2;21(9):e0357452. doi: 10.1371/journal.pone.0357452 (PMC13537581; doi:10.1371/journal.pone.0357452)
Supplement: Supplementary file 2 [file pone.0357452.s002.docx]

**S2 Table:** Trait data (BLUEs) for the half-sib families in Lamberton, MN and St. Paul, MN in 2021, 2022, and 2023.

| **ID** | **Mother** | **Env** | **Height_BLUE** | **Yield_BLUE** | **TKW_BLUE** |
| --- | --- | --- | --- | --- | --- |
| C4HSF001 | C418S0213 | Lam21 | 124.25 | 38.54 | 7.58 |
| C4HSF002 | C418S0228 | Lam21 | 127.00 | 23.28 | 6.30 |
| C4HSF003 | C418S0252 | Lam21 | 139.25 | 24.34 | 7.61 |
| C4HSF004 | C418S0263 | Lam21 | 142.75 | 38.51 | 8.49 |
| C4HSF005 | C418S0284 | Lam21 | 127.50 | 39.66 | 8.17 |
| C4HSF006 | C418S0286 | Lam21 | 141.50 | 30.91 | 7.72 |
| C4HSF007 | C418S0425 | Lam21 | 129.25 | 23.57 | 5.75 |
| C4HSF008 | C418S0469 | Lam21 | 132.50 | 32.35 | 7.45 |
| C4HSF009 | C418S0499 | Lam21 | 126.00 | 22.77 | 7.56 |
| C4HSF010 | C418S0565 | Lam21 | 128.50 | 27.46 | 6.42 |
| C4HSF011 | C418S0619 | Lam21 | 135.50 | 33.76 | 7.49 |
| C4HSF012 | C418S0003 | Lam21 | 125.25 | 32.13 | 7.44 |
| C4HSF013 | C418S0004 | Lam21 | 124.00 | 37.08 | 7.38 |
| C4HSF014 | C418S0006 | Lam21 | 129.25 | 31.09 | 8.11 |
| C4HSF015 | C418S0007 | Lam21 | 122.50 | 34.08 | 7.12 |
| C4HSF016 | C418S0008 | Lam21 | 139.25 | 28.92 | 7.49 |
| C4HSF017 | C418S0009 | Lam21 | 145.50 | 36.80 | 6.82 |
| C4HSF018 | C418S0010 | Lam21 | 128.25 | 20.45 | 6.85 |
| C4HSF019 | C418S0014 | Lam21 | 126.25 | 16.74 | 6.40 |
| C4HSF020 | C418S0015 | Lam21 | 126.00 | 36.56 | 8.02 |
| C4HSF021 | C418S0017 | Lam21 | 137.75 | 17.45 | 8.17 |
| C4HSF022 | C418S0020 | Lam21 | 128.75 | 27.66 | 7.17 |
| C4HSF023 | C418S0021 | Lam21 | 128.75 | 24.08 | 7.09 |
| C4HSF024 | C418S0022 | Lam21 | 127.75 | 37.05 | 8.66 |
| C4HSF025 | C418S0023 | Lam21 | 119.25 | 28.92 | 6.90 |
| C4HSF026 | C418S0024 | Lam21 | 135.75 | 35.34 | 7.73 |
| C4HSF027 | C418S0025 | Lam21 | 133.75 | 30.36 | 7.28 |
| C4HSF028 | C418S0026 | Lam21 | 131.50 | 46.11 | 8.84 |
| C4HSF029 | C418S0028 | Lam21 | 128.00 | 21.68 | 7.03 |
| C4HSF030 | C418S0031 | Lam21 | 133.75 | 16.01 | 6.32 |
| C4HSF031 | C418S0032 | Lam21 | 136.25 | 29.41 | 8.34 |
| C4HSF032 | C418S0034 | Lam21 | 124.75 | 28.61 | 7.86 |
| C4HSF033 | C418S0035 | Lam21 | 138.25 | 26.49 | 7.17 |
| C4HSF034 | C418S0051 | Lam21 | 138.75 | 37.12 | 6.96 |
| C4HSF035 | C418S0054 | Lam21 | 135.75 | 37.56 | 6.62 |
| C4HSF036 | C418S0056 | Lam21 | 135.25 | 39.61 | 7.66 |
| C4HSF037 | C418S0061 | Lam21 | 130.00 | 38.15 | 7.65 |
| C4HSF038 | C418S0062 | Lam21 | 136.25 | 30.41 | 8.32 |
| C4HSF039 | C418S0063 | Lam21 | 127.50 | 33.37 | 6.74 |
| C4HSF040 | C418S0068 | Lam21 | 133.25 | 36.57 | 7.66 |
| C4HSF041 | C418S0079 | Lam21 | 139.00 | 36.66 | 8.24 |
| C4HSF042 | C418S0084 | Lam21 | 127.25 | 40.23 | 7.26 |
| C4HSF043 | C418S0086 | Lam21 | 130.75 | 33.58 | 8.68 |
| C4HSF044 | C418S0087 | Lam21 | 128.25 | 48.35 | 8.10 |
| C4HSF045 | C418S0089 | Lam21 | 131.25 | 38.26 | 9.11 |
| C4HSF046 | C418S0092 | Lam21 | 135.50 | 29.32 | 6.80 |
| C4HSF047 | C418S0093 | Lam21 | 137.00 | 37.63 | 5.88 |
| C4HSF049 | C418S0097 | Lam21 | 133.00 | 22.40 | 7.64 |
| C4HSF050 | C418S0098 | Lam21 | 134.25 | 30.20 | 6.65 |
| C4HSF051 | C418S0100 | Lam21 | 133.00 | 32.23 | 8.76 |
| C4HSF052 | C418S0103 | Lam21 | 134.25 | 31.37 | 8.57 |
| C4HSF054 | C418S0118 | Lam21 | 141.50 | 31.70 | 6.91 |
| C4HSF055 | C418S0119 | Lam21 | 131.00 | 37.12 | 7.45 |
| C4HSF056 | C418S0120 | Lam21 | 131.25 | 30.48 | 6.01 |
| C4HSF057 | C418S0121 | Lam21 | 126.50 | 28.61 | 7.17 |
| C4HSF058 | C418S0122 | Lam21 | 134.00 | 26.73 | 8.50 |
| C4HSF059 | C418S0124 | Lam21 | 127.25 | 30.30 | 7.75 |
| C4HSF060 | C418S0125 | Lam21 | 123.50 | 26.14 | 6.44 |
| C4HSF061 | C418S0126 | Lam21 | 137.00 | 33.66 | 9.57 |
| C4HSF062 | C418S0128 | Lam21 | 130.00 | 24.96 | 6.86 |
| C4HSF063 | C418S0129 | Lam21 | 138.50 | 31.17 | 7.25 |
| C4HSF064 | C418S0131 | Lam21 | 131.50 | 43.97 | 7.21 |
| C4HSF065 | C418S0133 | Lam21 | 127.75 | 36.04 | 6.70 |
| C4HSF066 | C418S0134 | Lam21 | 136.50 | 27.88 | 7.56 |
| C4HSF067 | C418S0135 | Lam21 | 125.75 | 25.53 | 6.57 |
| C4HSF068 | C418S0137 | Lam21 | 129.00 | 37.27 | 8.28 |
| C4HSF069 | C418S0138 | Lam21 | 137.50 | 32.30 | 6.78 |
| C4HSF070 | C418S0139 | Lam21 | 129.25 | 27.69 | 9.04 |
| C4HSF071 | C418S0140 | Lam21 | 117.25 | 32.64 | 8.55 |
| C4HSF073 | C418S0143 | Lam21 | 138.00 | 23.58 | 8.72 |
| C4HSF074 | C418S0145 | Lam21 | 128.00 | 38.52 | 7.04 |
| C4HSF076 | C418S0149 | Lam21 | 134.00 | 20.96 | 7.00 |
| C4HSF077 | C418S0150 | Lam21 | 133.50 | 22.04 | 8.54 |
| C4HSF078 | C418S0151 | Lam21 | 123.00 | 28.52 | 7.16 |
| C4HSF079 | C418S0152 | Lam21 | 144.25 | 28.55 | 9.05 |
| C4HSF080 | C418S0153 | Lam21 | 135.75 | 28.63 | 6.26 |
| C4HSF081 | C418S0154 | Lam21 | 146.00 | 16.98 | 8.22 |
| C4HSF082 | C418S0156 | Lam21 | 131.00 | 20.06 | 8.99 |
| C4HSF083 | C418S0157 | Lam21 | 135.00 | 35.95 | 8.36 |
| C4HSF084 | C418S0161 | Lam21 | 138.50 | 43.68 | 7.49 |
| C4HSF085 | C418S0163 | Lam21 | 132.50 | 30.77 | 6.83 |
| C4HSF086 | C418S0164 | Lam21 | 134.25 | 35.52 | 9.54 |
| C4HSF087 | C418S0165 | Lam21 | 136.75 | 37.22 | 8.19 |
| C4HSF088 | C418S0166 | Lam21 | 130.75 | 28.85 | 8.08 |
| C4HSF089 | C418S0167 | Lam21 | 142.00 | 38.60 | 6.62 |
| C4HSF090 | C418S0168 | Lam21 | 135.25 | 29.62 | 7.49 |
| C4HSF091 | C418S0169 | Lam21 | 132.75 | 50.11 | 8.20 |
| C4HSF092 | C418S0171 | Lam21 | 115.00 | 36.46 | 9.31 |
| C4HSF093 | C418S0172 | Lam21 | 128.50 | 22.34 | 8.88 |
| C4HSF094 | C418S0177 | Lam21 | 138.75 | 29.50 | 6.76 |
| C4HSF095 | C418S0178 | Lam21 | 136.50 | 28.90 | 7.34 |
| C4HSF096 | C418S0180 | Lam21 | 122.25 | 30.52 | 8.00 |
| C4HSF097 | C418S0193 | Lam21 | 140.50 | 40.41 | 7.13 |
| C4HSF098 | C418S0196 | Lam21 | 142.00 | 27.58 | 6.64 |
| C4HSF099 | C418S0197 | Lam21 | 138.50 | 29.13 | 7.19 |
| C4HSF100 | C418S0199 | Lam21 | 141.75 | 26.87 | 6.66 |
| C4HSF101 | C418S0201 | Lam21 | 132.75 | 23.17 | 7.51 |
| C4HSF102 | C418S0202 | Lam21 | 131.00 | 22.83 | 7.95 |
| C4HSF103 | C418S0206 | Lam21 | 117.25 | 25.84 | 9.43 |
| C4HSF104 | C418S0207 | Lam21 | 123.25 | 29.16 | 8.06 |
| C4HSF105 | C418S0208 | Lam21 | 132.50 | 25.55 | 8.10 |
| C4HSF106 | C418S0209 | Lam21 | 127.75 | 21.24 | 7.43 |
| C4HSF107 | C418S0210 | Lam21 | 145.00 | 38.54 | 8.47 |
| C4HSF108 | C418S0215 | Lam21 | 137.25 | 31.65 | 6.67 |
| C4HSF109 | C418S0217 | Lam21 | 135.50 | 40.95 | 7.21 |
| C4HSF110 | C418S0219 | Lam21 | 137.50 | 28.36 | 8.05 |
| C4HSF111 | C418S0220 | Lam21 | 133.75 | 30.33 | 7.56 |
| C4HSF112 | C418S0225 | Lam21 | 130.50 | 25.10 | 7.08 |
| C4HSF113 | C418S0226 | Lam21 | 132.00 | 31.90 | 6.55 |
| C4HSF114 | C418S0230 | Lam21 | 137.25 | 23.48 | 8.23 |
| C4HSF115 | C418S0232 | Lam21 | 132.50 | 29.34 | 8.47 |
| C4HSF116 | C418S0233 | Lam21 | 140.00 | 21.63 | 6.96 |
| C4HSF117 | C418S0234 | Lam21 | 129.75 | 31.71 | 8.46 |
| C4HSF118 | C418S0235 | Lam21 | 131.25 | 24.08 | 8.54 |
| C4HSF119 | C418S0236 | Lam21 | 128.50 | 28.50 | 8.47 |
| C4HSF120 | C418S0237 | Lam21 | 128.25 | 32.62 | 7.40 |
| C4HSF121 | C418S0240 | Lam21 | 113.75 | 37.35 | 7.20 |
| C4HSF122 | C418S0242 | Lam21 | 133.50 | 31.55 | 8.50 |
| C4HSF123 | C418S0243 | Lam21 | 132.75 | 21.34 | 6.56 |
| C4HSF124 | C418S0244 | Lam21 | 141.50 | 21.86 | 6.76 |
| C4HSF125 | C418S0245 | Lam21 | 120.00 | 32.07 | 7.39 |
| C4HSF126 | C418S0253 | Lam21 | 133.75 | 23.30 | 7.59 |
| C4HSF127 | C418S0254 | Lam21 | 123.25 | 23.93 | 7.21 |
| C4HSF128 | C418S0259 | Lam21 | 136.75 | 33.27 | 7.41 |
| C4HSF129 | C418S0260 | Lam21 | 136.25 | 26.50 | 7.33 |
| C4HSF130 | C418S0264 | Lam21 | 133.50 | 26.05 | 8.13 |
| C4HSF131 | C418S0267 | Lam21 | 133.25 | 28.76 | 6.71 |
| C4HSF132 | C418S0268 | Lam21 | 144.75 | 33.21 | 7.81 |
| C4HSF133 | C418S0269 | Lam21 | 138.25 | 31.66 | 6.75 |
| C4HSF134 | C418S0270 | Lam21 | 118.00 | 30.95 | 7.30 |
| C4HSF135 | C418S0273 | Lam21 | 126.50 | 30.49 | 6.18 |
| C4HSF136 | C418S0274 | Lam21 | 128.50 | 32.75 | 8.61 |
| C4HSF137 | C418S0276 | Lam21 | 125.25 | 35.67 | 8.28 |
| C4HSF138 | C418S0278 | Lam21 | 133.25 | 31.80 | 7.12 |
| C4HSF139 | C418S0280 | Lam21 | 143.00 | 28.37 | 6.93 |
| C4HSF140 | C418S0281 | Lam21 | 135.50 | 34.90 | 7.25 |
| C4HSF141 | C418S0282 | Lam21 | 129.75 | 34.75 | 7.69 |
| C4HSF142 | C418S0283 | Lam21 | 129.75 | 31.19 | 7.81 |
| C4HSF143 | C418S0285 | Lam21 | 133.50 | 33.25 | 9.09 |
| C4HSF144 | C418S0289 | Lam21 | 123.00 | 20.28 | 7.49 |
| C4HSF145 | C418S0291 | Lam21 | 120.50 | 22.40 | 6.81 |
| C4HSF146 | C418S0298 | Lam21 | 136.25 | 28.33 | 6.79 |
| C4HSF147 | C418S0301 | Lam21 | 130.50 | 30.53 | 6.58 |
| C4HSF148 | C418S0302 | Lam21 | 138.25 | 40.06 | 6.60 |
| C4HSF149 | C418S0303 | Lam21 | 128.50 | 35.77 | 6.76 |
| C4HSF150 | C418S0304 | Lam21 | 127.00 | 42.73 | 7.67 |
| C4HSF151 | C418S0305 | Lam21 | 133.25 | 36.40 | 7.89 |
| C4HSF152 | C418S0308 | Lam21 | 124.00 | 26.15 | 6.82 |
| C4HSF153 | C418S0309 | Lam21 | 126.25 | 34.26 | 7.94 |
| C4HSF154 | C418S0315 | Lam21 | 134.50 | 39.19 | 8.23 |
| C4HSF155 | C418S0321 | Lam21 | 126.50 | 29.65 | 6.67 |
| C4HSF156 | C418S0325 | Lam21 | 126.00 | 41.79 | 8.47 |
| C4HSF157 | C418S0327 | Lam21 | 122.75 | 25.06 | 7.65 |
| C4HSF158 | C418S0330 | Lam21 | 139.25 | 38.22 | 6.48 |
| C4HSF159 | C418S0336 | Lam21 | 130.25 | 37.57 | 6.64 |
| C4HSF160 | C418S0337 | Lam21 | 122.50 | 20.85 | 6.47 |
| C4HSF161 | C418S0340 | Lam21 | 124.75 | 25.93 | 8.04 |
| C4HSF162 | C418S0343 | Lam21 | 135.25 | 17.20 | 7.20 |
| C4HSF163 | C418S0344 | Lam21 | 133.75 | 27.95 | 7.93 |
| C4HSF164 | C418S0350 | Lam21 | 134.50 | 28.37 | 6.54 |
| C4HSF165 | C418S0351 | Lam21 | 135.00 | 33.21 | 8.14 |
| C4HSF166 | C418S0352 | Lam21 | 134.50 | 25.73 | 6.81 |
| C4HSF167 | C418S0353 | Lam21 | 130.50 | 26.85 | 8.19 |
| C4HSF168 | C418S0355 | Lam21 | 137.00 | 26.13 | 7.68 |
| C4HSF169 | C418S0356 | Lam21 | 128.50 | 30.08 | 7.33 |
| C4HSF170 | C418S0357 | Lam21 | 132.25 | 31.40 | 8.31 |
| C4HSF171 | C418S0358 | Lam21 | 118.50 | 27.76 | 6.85 |
| C4HSF172 | C418S0360 | Lam21 | 124.00 | 21.12 | 7.07 |
| C4HSF173 | C418S0362 | Lam21 | 120.00 | 24.34 | 7.72 |
| C4HSF174 | C418S0364 | Lam21 | 138.00 | 42.52 | 7.90 |
| C4HSF175 | C418S0365 | Lam21 | 130.50 | 30.33 | 7.22 |
| C4HSF176 | C418S0367 | Lam21 | 129.75 | 27.23 | 8.16 |
| C4HSF177 | C418S0368 | Lam21 | 122.50 | 32.62 | 7.96 |
| C4HSF178 | C418S0369 | Lam21 | 136.00 | 34.77 | 7.49 |
| C4HSF179 | C418S0382 | Lam21 | 140.50 | 24.87 | 8.35 |
| C4HSF180 | C418S0385 | Lam21 | 126.75 | 25.23 | 7.52 |
| C4HSF181 | C418S0386 | Lam21 | 138.25 | 38.58 | 8.78 |
| C4HSF182 | C418S0390 | Lam21 | 138.50 | 39.80 | 8.74 |
| C4HSF183 | C418S0391 | Lam21 | 142.00 | 39.61 | 7.91 |
| C4HSF184 | C418S0392 | Lam21 | 132.00 | 28.01 | 6.93 |
| C4HSF185 | C418S0394 | Lam21 | 134.00 | 24.83 | 6.26 |
| C4HSF186 | C418S0396 | Lam21 | 133.00 | 33.19 | 8.28 |
| C4HSF187 | C418S0399 | Lam21 | 132.25 | 27.10 | 6.46 |
| C4HSF188 | C418S0401 | Lam21 | 129.50 | 26.40 | 7.11 |
| C4HSF189 | C418S0406 | Lam21 | 134.25 | 26.02 | 7.03 |
| C4HSF190 | C418S0410 | Lam21 | 133.75 | 27.17 | 7.39 |
| C4HSF191 | C418S0411 | Lam21 | 123.25 | 33.07 | 6.67 |
| C4HSF192 | C418S0412 | Lam21 | 134.00 | 40.02 | 7.13 |
| C4HSF193 | C418S0413 | Lam21 | 122.50 | 26.67 | 6.85 |
| C4HSF194 | C418S0414 | Lam21 | 129.00 | 33.00 | 8.30 |
| C4HSF195 | C418S0420 | Lam21 | 140.50 | 35.85 | 6.62 |
| C4HSF196 | C418S0421 | Lam21 | 133.50 | 26.75 | 7.30 |
| C4HSF197 | C418S0422 | Lam21 | 133.75 | 35.43 | 7.59 |
| C4HSF198 | C418S0423 | Lam21 | 125.25 | 23.16 | 8.41 |
| C4HSF199 | C418S0426 | Lam21 | 139.50 | 46.48 | 6.48 |
| C4HSF200 | C418S0427 | Lam21 | 135.25 | 24.57 | 7.17 |
| C4HSF201 | C418S0428 | Lam21 | 140.00 | 44.78 | 7.01 |
| C4HSF202 | C418S0429 | Lam21 | 132.00 | 30.44 | 7.18 |
| C4HSF203 | C418S0430 | Lam21 | 137.25 | 50.55 | 7.65 |
| C4HSF204 | C418S0455 | Lam21 | 133.50 | 35.05 | 9.11 |
| C4HSF205 | C418S0458 | Lam21 | 126.67 | 25.34 | 8.13 |
| C4HSF206 | C418S0463 | Lam21 | 130.00 | 38.45 | 7.65 |
| C4HSF211 | C418S0489 | Lam21 | 141.50 | 44.90 | 8.02 |
| C4HSF212 | C418S0491 | Lam21 | 135.50 | 32.52 | 8.00 |
| C4HSF213 | C418S0492 | Lam21 | 124.00 | 36.28 | 7.44 |
| C4HSF217 | C418S0497 | Lam21 | 134.50 | 44.57 | 6.92 |
| C4HSF223 | C418S0572 | Lam21 | 127.75 | 22.69 | 7.96 |
| C4HSF001 | C418S0213 | Lam22 | 128.06 | 33.98 | 7.77 |
| C4HSF002 | C418S0228 | Lam22 | 123.81 | 35.37 | 6.85 |
| C4HSF003 | C418S0252 | Lam22 | 136.81 | 31.97 | 6.95 |
| C4HSF004 | C418S0263 | Lam22 | 139.06 | 40.60 | 7.17 |
| C4HSF005 | C418S0284 | Lam22 | 131.81 | 24.30 | 7.53 |
| C4HSF006 | C418S0286 | Lam22 | 135.06 | 30.61 | 7.65 |
| C4HSF007 | C418S0425 | Lam22 | 132.06 | 27.43 | 8.78 |
| C4HSF008 | C418S0469 | Lam22 | 135.81 | 28.18 | 7.55 |
| C4HSF009 | C418S0499 | Lam22 | 130.06 | 41.63 | 7.39 |
| C4HSF010 | C418S0565 | Lam22 | 128.56 | 26.66 | 7.43 |
| C4HSF011 | C418S0619 | Lam22 | 133.06 | 30.29 | 8.39 |
| C4HSF012 | C418S0003 | Lam22 | 128.06 | 33.82 | 7.89 |
| C4HSF013 | C418S0004 | Lam22 | 129.81 | 29.63 | 7.29 |
| C4HSF014 | C418S0006 | Lam22 | 135.06 | 43.18 | 7.80 |
| C4HSF015 | C418S0007 | Lam22 | 129.06 | 36.30 | 8.26 |
| C4HSF016 | C418S0008 | Lam22 | 134.31 | 38.81 | 8.49 |
| C4HSF017 | C418S0009 | Lam22 | 138.81 | 23.44 | 7.20 |
| C4HSF018 | C418S0010 | Lam22 | 131.31 | 23.01 | 7.56 |
| C4HSF019 | C418S0014 | Lam22 | 128.06 | 36.57 | 7.24 |
| C4HSF020 | C418S0015 | Lam22 | 130.56 | 26.20 | 8.51 |
| C4HSF021 | C418S0017 | Lam22 | 134.31 | 31.03 | 7.75 |
| C4HSF022 | C418S0020 | Lam22 | 138.31 | 25.98 | 6.99 |
| C4HSF023 | C418S0021 | Lam22 | 136.81 | 23.35 | 7.48 |
| C4HSF024 | C418S0022 | Lam22 | 128.06 | 34.55 | 7.48 |
| C4HSF025 | C418S0023 | Lam22 | 135.06 | 23.15 | 7.96 |
| C4HSF026 | C418S0024 | Lam22 | 135.56 | 21.16 | 7.76 |
| C4HSF027 | C418S0025 | Lam22 | 134.31 | 34.75 | 7.18 |
| C4HSF028 | C418S0026 | Lam22 | 132.56 | 24.74 | 7.87 |
| C4HSF029 | C418S0028 | Lam22 | 130.06 | 27.76 | 7.59 |
| C4HSF030 | C418S0031 | Lam22 | 133.06 | 29.58 | 8.50 |
| C4HSF031 | C418S0032 | Lam22 | 135.31 | 34.20 | 7.36 |
| C4HSF032 | C418S0034 | Lam22 | 126.81 | 30.34 | 7.74 |
| C4HSF033 | C418S0035 | Lam22 | 131.81 | 21.18 | 7.44 |
| C4HSF034 | C418S0051 | Lam22 | 134.81 | 31.18 | 6.79 |
| C4HSF035 | C418S0054 | Lam22 | 135.06 | 36.59 | 6.36 |
| C4HSF036 | C418S0056 | Lam22 | 139.56 | 22.49 | 7.58 |
| C4HSF037 | C418S0061 | Lam22 | 127.56 | 25.10 | 6.79 |
| C4HSF038 | C418S0062 | Lam22 | 134.81 | 47.46 | 6.56 |
| C4HSF039 | C418S0063 | Lam22 | 124.81 | 29.90 | 6.41 |
| C4HSF040 | C418S0068 | Lam22 | 136.31 | 24.19 | 6.65 |
| C4HSF041 | C418S0079 | Lam22 | 128.06 | 33.11 | 8.00 |
| C4HSF042 | C418S0084 | Lam22 | 127.81 | 30.56 | 7.46 |
| C4HSF043 | C418S0086 | Lam22 | 132.56 | 42.78 | 7.81 |
| C4HSF044 | C418S0087 | Lam22 | 136.56 | 25.58 | 7.68 |
| C4HSF045 | C418S0089 | Lam22 | 131.56 | 31.76 | 7.92 |
| C4HSF046 | C418S0092 | Lam22 | 137.06 | 26.82 | 7.94 |
| C4HSF047 | C418S0093 | Lam22 | 129.81 | 45.25 | 8.27 |
| C4HSF049 | C418S0097 | Lam22 | 131.31 | 40.57 | 8.33 |
| C4HSF050 | C418S0098 | Lam22 | 136.81 | 26.28 | 7.06 |
| C4HSF051 | C418S0100 | Lam22 | 136.56 | 33.01 | 7.00 |
| C4HSF052 | C418S0103 | Lam22 | 128.56 | 39.08 | 6.96 |
| C4HSF054 | C418S0118 | Lam22 | 136.06 | 31.90 | 7.60 |
| C4HSF055 | C418S0119 | Lam22 | 132.56 | 32.44 | 6.23 |
| C4HSF056 | C418S0120 | Lam22 | 132.31 | 30.34 | 7.99 |
| C4HSF057 | C418S0121 | Lam22 | 128.06 | 34.87 | 6.85 |
| C4HSF058 | C418S0122 | Lam22 | 129.31 | 29.39 | 7.23 |
| C4HSF059 | C418S0124 | Lam22 | 65.06 | 36.49 | 7.01 |
| C4HSF060 | C418S0125 | Lam22 | 129.56 | 48.29 | 7.05 |
| C4HSF061 | C418S0126 | Lam22 | 134.31 | 28.98 | 7.45 |
| C4HSF062 | C418S0128 | Lam22 | 135.81 | 35.58 | 6.63 |
| C4HSF063 | C418S0129 | Lam22 | 137.06 | 29.96 | 7.49 |
| C4HSF064 | C418S0131 | Lam22 | 130.56 | 40.38 | 7.57 |
| C4HSF065 | C418S0133 | Lam22 | 125.81 | 46.60 | 7.55 |
| C4HSF066 | C418S0134 | Lam22 | 133.31 | 27.65 | 6.92 |
| C4HSF067 | C418S0135 | Lam22 | 135.06 | 28.62 | 7.25 |
| C4HSF068 | C418S0137 | Lam22 | 129.56 | 29.38 | 7.76 |
| C4HSF069 | C418S0138 | Lam22 | 132.06 | 33.27 | 8.25 |
| C4HSF070 | C418S0139 | Lam22 | 130.56 | 33.06 | 7.76 |
| C4HSF071 | C418S0140 | Lam22 | 124.31 | 28.74 | 7.09 |
| C4HSF073 | C418S0143 | Lam22 | 133.81 | 24.21 | 7.67 |
| C4HSF074 | C418S0145 | Lam22 | 131.56 | 32.33 | 7.51 |
| C4HSF076 | C418S0149 | Lam22 | 127.31 | 25.90 | 8.23 |
| C4HSF077 | C418S0150 | Lam22 | 133.81 | 29.02 | 7.53 |
| C4HSF078 | C418S0151 | Lam22 | 137.81 | 31.53 | 6.77 |
| C4HSF079 | C418S0152 | Lam22 | 138.31 | 30.41 | 7.02 |
| C4HSF080 | C418S0153 | Lam22 | 129.81 | 27.44 | 6.80 |
| C4HSF081 | C418S0154 | Lam22 | 134.81 | 20.82 | 7.11 |
| C4HSF082 | C418S0156 | Lam22 | 132.06 | 24.27 | 7.55 |
| C4HSF083 | C418S0157 | Lam22 | 136.31 | 30.32 | 7.61 |
| C4HSF084 | C418S0161 | Lam22 | 133.81 | 31.45 | 7.51 |
| C4HSF085 | C418S0163 | Lam22 | 134.31 | 27.04 | 7.88 |
| C4HSF086 | C418S0164 | Lam22 | 136.31 | 35.49 | 7.49 |
| C4HSF087 | C418S0165 | Lam22 | 132.31 | 25.77 | 7.03 |
| C4HSF088 | C418S0166 | Lam22 | 137.56 | 31.59 | 8.48 |
| C4HSF089 | C418S0167 | Lam22 | 134.56 | 27.51 | 7.64 |
| C4HSF090 | C418S0168 | Lam22 | 139.56 | 32.71 | 8.24 |
| C4HSF091 | C418S0169 | Lam22 | 131.81 | 25.85 | 8.11 |
| C4HSF092 | C418S0171 | Lam22 | 130.31 | 25.20 | 7.45 |
| C4HSF093 | C418S0172 | Lam22 | 135.81 | 22.41 | 7.92 |
| C4HSF094 | C418S0177 | Lam22 | 135.31 | 25.87 | 8.10 |
| C4HSF095 | C418S0178 | Lam22 | 133.06 | 26.56 | 6.88 |
| C4HSF096 | C418S0180 | Lam22 | 138.31 | 39.86 | 7.26 |
| C4HSF097 | C418S0193 | Lam22 | 132.81 | 33.73 | 7.00 |
| C4HSF098 | C418S0196 | Lam22 | 134.31 | 25.20 | 7.05 |
| C4HSF099 | C418S0197 | Lam22 | 138.06 | 39.03 | 8.31 |
| C4HSF100 | C418S0199 | Lam22 | 136.31 | 31.35 | 6.80 |
| C4HSF101 | C418S0201 | Lam22 | 134.06 | 41.36 | 6.96 |
| C4HSF102 | C418S0202 | Lam22 | 130.81 | 42.76 | 7.82 |
| C4HSF103 | C418S0206 | Lam22 | 122.81 | 30.37 | 7.54 |
| C4HSF104 | C418S0207 | Lam22 | 130.06 | 19.32 | 8.04 |
| C4HSF105 | C418S0208 | Lam22 | 133.31 | 28.28 | 7.36 |
| C4HSF106 | C418S0209 | Lam22 | 130.81 | 20.53 | 6.94 |
| C4HSF107 | C418S0210 | Lam22 | 133.31 | 31.90 | 7.45 |
| C4HSF108 | C418S0215 | Lam22 | 136.56 | 41.95 | 7.70 |
| C4HSF109 | C418S0217 | Lam22 | 136.56 | 29.03 | 8.13 |
| C4HSF110 | C418S0219 | Lam22 | 135.31 | 41.91 | 7.05 |
| C4HSF111 | C418S0220 | Lam22 | 134.06 | 24.92 | 7.51 |
| C4HSF112 | C418S0225 | Lam22 | 131.06 | 28.31 | 7.79 |
| C4HSF113 | C418S0226 | Lam22 | 132.56 | 30.64 | 7.60 |
| C4HSF114 | C418S0230 | Lam22 | 135.06 | 33.93 | 7.78 |
| C4HSF115 | C418S0232 | Lam22 | 136.06 | 25.82 | 7.81 |
| C4HSF116 | C418S0233 | Lam22 | 140.56 | 38.29 | 7.08 |
| C4HSF117 | C418S0234 | Lam22 | 130.06 | 38.75 | 6.74 |
| C4HSF118 | C418S0235 | Lam22 | 133.56 | 24.61 | 7.91 |
| C4HSF119 | C418S0236 | Lam22 | 130.56 | 28.79 | 7.39 |
| C4HSF120 | C418S0237 | Lam22 | 133.31 | 28.23 | 7.19 |
| C4HSF121 | C418S0240 | Lam22 | 130.06 | 22.22 | 8.31 |
| C4HSF122 | C418S0242 | Lam22 | 132.81 | 22.08 | 7.12 |
| C4HSF123 | C418S0243 | Lam22 | 135.06 | 31.82 | 6.96 |
| C4HSF124 | C418S0244 | Lam22 | 140.31 | 32.12 | 7.51 |
| C4HSF125 | C418S0245 | Lam22 | 130.31 | 29.31 | 8.12 |
| C4HSF126 | C418S0253 | Lam22 | 129.81 | 25.55 | 7.60 |
| C4HSF127 | C418S0254 | Lam22 | 133.31 | 41.33 | 7.52 |
| C4HSF128 | C418S0259 | Lam22 | 135.06 | 26.47 | 6.80 |
| C4HSF129 | C418S0260 | Lam22 | 135.06 | 24.57 | 7.28 |
| C4HSF130 | C418S0264 | Lam22 | 133.56 | 29.15 | 7.60 |
| C4HSF131 | C418S0267 | Lam22 | 132.31 | 34.50 | 8.30 |
| C4HSF132 | C418S0268 | Lam22 | 139.56 | 35.46 | 6.60 |
| C4HSF133 | C418S0269 | Lam22 | 136.56 | 44.15 | 9.17 |
| C4HSF134 | C418S0270 | Lam22 | 134.31 | 30.20 | 7.45 |
| C4HSF135 | C418S0273 | Lam22 | 132.06 | 20.55 | 6.91 |
| C4HSF136 | C418S0274 | Lam22 | 134.31 | 22.93 | 7.77 |
| C4HSF137 | C418S0276 | Lam22 | 131.31 | 27.52 | 7.46 |
| C4HSF138 | C418S0278 | Lam22 | 133.31 | 27.09 | 7.38 |
| C4HSF139 | C418S0280 | Lam22 | 133.31 | 30.73 | 6.78 |
| C4HSF140 | C418S0281 | Lam22 | 131.56 | 27.47 | 8.54 |
| C4HSF141 | C418S0282 | Lam22 | 130.06 | 27.11 | 6.74 |
| C4HSF142 | C418S0283 | Lam22 | 132.56 | 43.19 | 7.01 |
| C4HSF143 | C418S0285 | Lam22 | 134.06 | 27.42 | 8.35 |
| C4HSF144 | C418S0289 | Lam22 | 129.06 | 30.32 | 7.89 |
| C4HSF145 | C418S0291 | Lam22 | 137.06 | 24.19 | 7.43 |
| C4HSF146 | C418S0298 | Lam22 | 134.56 | 31.22 | 7.32 |
| C4HSF147 | C418S0301 | Lam22 | 136.81 | 22.68 | 7.79 |
| C4HSF148 | C418S0302 | Lam22 | 131.31 | 31.96 | 6.75 |
| C4HSF149 | C418S0303 | Lam22 | 132.81 | 33.97 | 7.17 |
| C4HSF150 | C418S0304 | Lam22 | 131.31 | 29.09 | 7.97 |
| C4HSF151 | C418S0305 | Lam22 | 127.81 | 31.33 | 7.42 |
| C4HSF152 | C418S0308 | Lam22 | 131.81 | 26.72 | 7.92 |
| C4HSF153 | C418S0309 | Lam22 | 132.56 | 25.87 | 8.71 |
| C4HSF154 | C418S0315 | Lam22 | 136.81 | 24.10 | 7.29 |
| C4HSF155 | C418S0321 | Lam22 | 134.31 | 26.68 | 7.44 |
| C4HSF156 | C418S0325 | Lam22 | 129.56 | 23.08 | 7.12 |
| C4HSF157 | C418S0327 | Lam22 | 132.31 | 28.15 | 7.38 |
| C4HSF158 | C418S0330 | Lam22 | 135.81 | 45.02 | 6.85 |
| C4HSF159 | C418S0336 | Lam22 | 130.06 | 19.99 | 7.21 |
| C4HSF160 | C418S0337 | Lam22 | 131.06 | 27.40 | 7.60 |
| C4HSF161 | C418S0340 | Lam22 | 133.81 | 48.91 | 7.40 |
| C4HSF162 | C418S0343 | Lam22 | 130.81 | 29.11 | 7.36 |
| C4HSF163 | C418S0344 | Lam22 | 125.56 | 32.45 | 7.12 |
| C4HSF164 | C418S0350 | Lam22 | 135.06 | 29.11 | 8.78 |
| C4HSF165 | C418S0351 | Lam22 | 132.56 | 33.53 | 7.64 |
| C4HSF166 | C418S0352 | Lam22 | 137.31 | 26.19 | 8.11 |
| C4HSF167 | C418S0353 | Lam22 | 127.06 | 30.30 | 7.33 |
| C4HSF168 | C418S0355 | Lam22 | 126.81 | 28.49 | 7.31 |
| C4HSF169 | C418S0356 | Lam22 | 131.06 | 25.92 | 7.00 |
| C4HSF170 | C418S0357 | Lam22 | 130.81 | 29.75 | 8.07 |
| C4HSF171 | C418S0358 | Lam22 | 132.56 | 37.51 | 6.69 |
| C4HSF172 | C418S0360 | Lam22 | 129.81 | 38.35 | 7.82 |
| C4HSF173 | C418S0362 | Lam22 | 129.06 | 23.41 | 7.55 |
| C4HSF174 | C418S0364 | Lam22 | 133.56 | 27.67 | 6.81 |
| C4HSF175 | C418S0365 | Lam22 | 131.06 | 38.56 | 8.34 |
| C4HSF176 | C418S0367 | Lam22 | 132.06 | 30.87 | 9.33 |
| C4HSF177 | C418S0368 | Lam22 | 125.81 | 20.70 | 7.93 |
| C4HSF178 | C418S0369 | Lam22 | 137.31 | 37.00 | 7.74 |
| C4HSF179 | C418S0382 | Lam22 | 133.06 | 33.75 | 7.14 |
| C4HSF180 | C418S0385 | Lam22 | 129.06 | 41.56 | 8.19 |
| C4HSF181 | C418S0386 | Lam22 | 134.06 | 36.76 | 7.47 |
| C4HSF182 | C418S0390 | Lam22 | 131.31 | 34.16 | 7.79 |
| C4HSF183 | C418S0391 | Lam22 | 136.56 | 33.93 | 7.52 |
| C4HSF184 | C418S0392 | Lam22 | 133.56 | 33.06 | 7.05 |
| C4HSF185 | C418S0394 | Lam22 | 66.81 | 30.30 | 7.36 |
| C4HSF186 | C418S0396 | Lam22 | 131.81 | 32.44 | 7.60 |
| C4HSF187 | C418S0399 | Lam22 | 134.81 | 33.07 | 6.67 |
| C4HSF188 | C418S0401 | Lam22 | 137.81 | 40.81 | 8.32 |
| C4HSF189 | C418S0406 | Lam22 | 128.56 | 23.41 | 7.29 |
| C4HSF190 | C418S0410 | Lam22 | 132.81 | 29.44 | 8.62 |
| C4HSF191 | C418S0411 | Lam22 | 137.81 | 30.74 | 7.24 |
| C4HSF192 | C418S0412 | Lam22 | 131.31 | 30.76 | 8.57 |
| C4HSF193 | C418S0413 | Lam22 | 126.81 | 25.42 | 7.26 |
| C4HSF194 | C418S0414 | Lam22 | 136.06 | 37.25 | 6.44 |
| C4HSF195 | C418S0420 | Lam22 | 137.56 | 29.34 | 8.33 |
| C4HSF196 | C418S0421 | Lam22 | 133.31 | 23.62 | 8.01 |
| C4HSF197 | C418S0422 | Lam22 | 132.31 | 26.58 | 7.15 |
| C4HSF198 | C418S0423 | Lam22 | 134.56 | 33.95 | 8.06 |
| C4HSF199 | C418S0426 | Lam22 | 128.56 | 43.72 | 7.72 |
| C4HSF200 | C418S0427 | Lam22 | 125.56 | 38.27 | 8.05 |
| C4HSF201 | C418S0428 | Lam22 | 141.81 | 39.51 | 8.84 |
| C4HSF202 | C418S0429 | Lam22 | 133.31 | 22.44 | 6.77 |
| C4HSF203 | C418S0430 | Lam22 | 134.56 | 29.85 | 7.82 |
| C4HSF204 | C418S0455 | Lam22 | 140.81 | 31.85 | 7.37 |
| C4HSF205 | C418S0458 | Lam22 | 134.14 | 26.13 | 7.36 |
| C4HSF206 | C418S0463 | Lam22 | 132.64 | 33.02 | 7.64 |
| C4HSF211 | C418S0489 | Lam22 | 135.31 | 36.45 | 7.76 |
| C4HSF212 | C418S0491 | Lam22 | 135.06 | 39.32 | 7.93 |
| C4HSF213 | C418S0492 | Lam22 | 68.31 | 33.63 | 7.63 |
| C4HSF217 | C418S0497 | Lam22 | 136.31 | 31.47 | 8.11 |
| C4HSF223 | C418S0572 | Lam22 | 124.56 | 27.90 | 8.71 |
| C4HSF001 | C418S0213 | StP21 | 137.05 | 27.77 | 7.29 |
| C4HSF002 | C418S0228 | StP21 | 123.80 | 32.92 | 7.45 |
| C4HSF003 | C418S0252 | StP21 | 142.55 | 29.18 | 7.08 |
| C4HSF004 | C418S0263 | StP21 | 129.80 | 34.44 | 8.00 |
| C4HSF005 | C418S0284 | StP21 | 133.55 | 25.49 | 7.84 |
| C4HSF006 | C418S0286 | StP21 | 136.05 | 30.90 | 7.48 |
| C4HSF007 | C418S0425 | StP21 | 134.05 | 29.04 | 7.28 |
| C4HSF008 | C418S0469 | StP21 | 126.05 | 24.89 | 7.69 |
| C4HSF009 | C418S0499 | StP21 | 135.80 | 39.30 | 7.87 |
| C4HSF010 | C418S0565 | StP21 | 139.05 | 21.04 | 7.11 |
| C4HSF011 | C418S0619 | StP21 | 125.30 | 42.06 | 7.59 |
| C4HSF012 | C418S0003 | StP21 | 131.05 | 48.59 | 7.25 |
| C4HSF013 | C418S0004 | StP21 | 119.30 | 22.69 | 9.44 |
| C4HSF014 | C418S0006 | StP21 | 137.80 | 32.13 | 7.70 |
| C4HSF015 | C418S0007 | StP21 | 130.30 | 22.66 | 5.74 |
| C4HSF016 | C418S0008 | StP21 | 137.80 | 31.01 | 7.13 |
| C4HSF017 | C418S0009 | StP21 | 123.80 | 28.49 | 6.52 |
| C4HSF018 | C418S0010 | StP21 | 137.30 | 28.43 | 8.17 |
| C4HSF019 | C418S0014 | StP21 | 131.97 | 39.49 | 8.41 |
| C4HSF020 | C418S0015 | StP21 | 124.80 | 37.72 | 8.50 |
| C4HSF021 | C418S0017 | StP21 | 125.55 | 28.83 | 6.66 |
| C4HSF022 | C418S0020 | StP21 | 130.05 | 38.34 | 7.08 |
| C4HSF023 | C418S0021 | StP21 | 134.80 | 19.57 | 7.31 |
| C4HSF024 | C418S0022 | StP21 | 141.55 | 34.01 | 9.30 |
| C4HSF025 | C418S0023 | StP21 | 136.55 | 43.85 | 8.85 |
| C4HSF026 | C418S0024 | StP21 | 128.05 | 33.50 | 8.07 |
| C4HSF027 | C418S0025 | StP21 | 142.30 | 24.23 | 7.04 |
| C4HSF028 | C418S0026 | StP21 | 136.80 | 34.26 | 6.80 |
| C4HSF029 | C418S0028 | StP21 | 120.05 | 26.18 | 7.05 |
| C4HSF030 | C418S0031 | StP21 | 129.64 | 23.13 | 7.65 |
| C4HSF031 | C418S0032 | StP21 | 125.30 | 21.11 | 7.98 |
| C4HSF032 | C418S0034 | StP21 | 130.55 | 27.66 | 7.11 |
| C4HSF033 | C418S0035 | StP21 | 131.30 | 21.01 | NA |
| C4HSF034 | C418S0051 | StP21 | 137.05 | 29.70 | 8.94 |
| C4HSF035 | C418S0054 | StP21 | 130.05 | 30.70 | 7.00 |
| C4HSF036 | C418S0056 | StP21 | 126.55 | 38.19 | 8.69 |
| C4HSF037 | C418S0061 | StP21 | 122.05 | 27.94 | 8.17 |
| C4HSF038 | C418S0062 | StP21 | 132.30 | 35.95 | 7.68 |
| C4HSF039 | C418S0063 | StP21 | 135.14 | 36.05 | 6.77 |
| C4HSF040 | C418S0068 | StP21 | 131.55 | 19.82 | 7.32 |
| C4HSF041 | C418S0079 | StP21 | 129.80 | 43.26 | 6.73 |
| C4HSF042 | C418S0084 | StP21 | 133.80 | 28.39 | 8.56 |
| C4HSF043 | C418S0086 | StP21 | 126.55 | 39.26 | 7.21 |
| C4HSF044 | C418S0087 | StP21 | 125.30 | 26.30 | 7.64 |
| C4HSF045 | C418S0089 | StP21 | 128.14 | 30.99 | 7.38 |
| C4HSF046 | C418S0092 | StP21 | 136.14 | 43.45 | 8.68 |
| C4HSF047 | C418S0093 | StP21 | 131.80 | 20.11 | 7.42 |
| C4HSF049 | C418S0097 | StP21 | 135.05 | 33.22 | NA |
| C4HSF050 | C418S0098 | StP21 | 126.30 | 31.63 | 7.51 |
| C4HSF051 | C418S0100 | StP21 | 132.30 | 33.86 | 6.54 |
| C4HSF052 | C418S0105 | StP21 | 135.80 | 26.45 | 7.20 |
| C4HSF054 | C418S0118 | StP21 | 135.30 | 43.82 | 8.92 |
| C4HSF055 | C418S0119 | StP21 | 135.05 | 23.12 | 7.20 |
| C4HSF056 | C418S0120 | StP21 | 130.80 | 26.35 | 8.09 |
| C4HSF057 | C418S0121 | StP21 | 131.30 | 34.80 | 7.11 |
| C4HSF058 | C418S0122 | StP21 | 137.05 | 37.56 | 7.73 |
| C4HSF059 | C418S0124 | StP21 | 134.55 | 31.92 | 8.29 |
| C4HSF060 | C418S0125 | StP21 | 125.30 | 32.15 | 7.33 |
| C4HSF061 | C418S0126 | StP21 | 136.55 | 31.44 | 7.66 |
| C4HSF062 | C418S0128 | StP21 | 139.55 | 23.66 | 7.19 |
| C4HSF063 | C418S0129 | StP21 | 130.80 | 23.49 | 6.52 |
| C4HSF064 | C418S0131 | StP21 | 129.80 | 14.60 | 7.92 |
| C4HSF065 | C418S0133 | StP21 | 131.05 | 39.16 | 7.26 |
| C4HSF066 | C418S0134 | StP21 | 131.30 | 40.98 | 9.07 |
| C4HSF067 | C418S0135 | StP21 | 132.05 | 24.13 | 6.40 |
| C4HSF068 | C418S0137 | StP21 | 127.30 | 28.35 | 7.45 |
| C4HSF069 | C418S0138 | StP21 | 138.47 | 37.23 | 7.49 |
| C4HSF070 | C418S0139 | StP21 | 125.30 | 24.85 | 7.29 |
| C4HSF071 | C418S0140 | StP21 | 142.30 | NA | NA |
| C4HSF073 | C418S0143 | StP21 | 123.80 | 30.67 | 7.78 |
| C4HSF074 | C418S0145 | StP21 | 127.55 | 28.56 | 7.06 |
| C4HSF076 | C418S0149 | StP21 | 145.80 | 28.74 | 7.84 |
| C4HSF077 | C418S0150 | StP21 | 135.80 | 25.39 | 7.55 |
| C4HSF078 | C418S0151 | StP21 | 127.05 | 31.49 | 8.70 |
| C4HSF079 | C418S0152 | StP21 | 124.30 | 26.55 | 7.07 |
| C4HSF080 | C418S0153 | StP21 | 147.55 | 19.68 | 7.88 |
| C4HSF081 | C418S0154 | StP21 | 139.05 | 30.82 | NA |
| C4HSF082 | C418S0156 | StP21 | 142.05 | 23.96 | 7.78 |
| C4HSF083 | C418S0157 | StP21 | 137.55 | 37.14 | 8.28 |
| C4HSF084 | C418S0161 | StP21 | 134.55 | 27.06 | 7.39 |
| C4HSF085 | C418S0163 | StP21 | 147.80 | 33.42 | 8.90 |
| C4HSF086 | C418S0164 | StP21 | 126.30 | 34.66 | 7.87 |
| C4HSF087 | C418S0165 | StP21 | 124.05 | 29.57 | 7.24 |
| C4HSF088 | C418S0166 | StP21 | 130.80 | 29.63 | 7.87 |
| C4HSF089 | C418S0167 | StP21 | 123.80 | 21.66 | 7.16 |
| C4HSF090 | C418S0168 | StP21 | 138.30 | 24.52 | 8.43 |
| C4HSF091 | C418S0169 | StP21 | 139.05 | 19.78 | 7.39 |
| C4HSF092 | C418S0171 | StP21 | 127.05 | 28.15 | 7.88 |
| C4HSF093 | C418S0172 | StP21 | 124.64 | 28.91 | 8.70 |
| C4HSF094 | C418S0177 | StP21 | 127.64 | 28.46 | 6.90 |
| C4HSF095 | C418S0178 | StP21 | 124.55 | 29.10 | 7.02 |
| C4HSF096 | C418S0180 | StP21 | 119.55 | 51.46 | 8.07 |
| C4HSF097 | C418S0193 | StP21 | 129.30 | 24.76 | 7.42 |
| C4HSF098 | C418S0196 | StP21 | 131.55 | 31.78 | 7.96 |
| C4HSF099 | C418S0197 | StP21 | 133.14 | 25.46 | 7.27 |
| C4HSF100 | C418S0199 | StP21 | 130.80 | 34.61 | 7.19 |
| C4HSF101 | C418S0201 | StP21 | 135.30 | 35.35 | 6.50 |
| C4HSF102 | C418S0202 | StP21 | 116.05 | 40.48 | 7.26 |
| C4HSF103 | C418S0206 | StP21 | 132.80 | 32.40 | 7.57 |
| C4HSF104 | C418S0207 | StP21 | 132.55 | 30.99 | 8.41 |
| C4HSF105 | C418S0208 | StP21 | 142.80 | 34.45 | 9.14 |
| C4HSF106 | C418S0209 | StP21 | 134.05 | 19.86 | 7.67 |
| C4HSF107 | C418S0210 | StP21 | 138.30 | 34.61 | 7.47 |
| C4HSF108 | C418S0215 | StP21 | 133.55 | 32.61 | 9.30 |
| C4HSF109 | C418S0217 | StP21 | 132.80 | 22.58 | 6.12 |
| C4HSF110 | C418S0219 | StP21 | 139.55 | 22.07 | 6.80 |
| C4HSF111 | C418S0220 | StP21 | 124.05 | 28.68 | 7.13 |
| C4HSF112 | C418S0225 | StP21 | 132.80 | 31.59 | 7.03 |
| C4HSF113 | C418S0226 | StP21 | 129.05 | 23.56 | 7.39 |
| C4HSF114 | C418S0230 | StP21 | 137.14 | 29.83 | 7.57 |
| C4HSF115 | C418S0232 | StP21 | 120.30 | 29.11 | 7.22 |
| C4HSF116 | C418S0233 | StP21 | 134.30 | 27.27 | 7.75 |
| C4HSF117 | C418S0234 | StP21 | 128.80 | 29.69 | 7.31 |
| C4HSF118 | C418S0235 | StP21 | 140.55 | 11.50 | 6.97 |
| C4HSF119 | C418S0236 | StP21 | 129.30 | 24.81 | 6.05 |
| C4HSF120 | C418S0237 | StP21 | 129.55 | 34.38 | 7.20 |
| C4HSF121 | C418S0240 | StP21 | 132.80 | 30.98 | 7.01 |
| C4HSF122 | C418S0242 | StP21 | 130.55 | 23.68 | 6.73 |
| C4HSF123 | C418S0243 | StP21 | 128.47 | 43.76 | 8.01 |
| C4HSF124 | C418S0244 | StP21 | 132.80 | 39.81 | 7.60 |
| C4HSF125 | C418S0245 | StP21 | 133.30 | 27.40 | 7.54 |
| C4HSF126 | C418S0253 | StP21 | 137.05 | 38.83 | 6.66 |
| C4HSF127 | C418S0254 | StP21 | 140.30 | 39.74 | 7.63 |
| C4HSF128 | C418S0259 | StP21 | 133.55 | 33.27 | 7.10 |
| C4HSF129 | C418S0260 | StP21 | 131.80 | 25.75 | 7.64 |
| C4HSF130 | C418S0264 | StP21 | 122.05 | 26.18 | 7.72 |
| C4HSF131 | C418S0267 | StP21 | 129.80 | 35.62 | 6.90 |
| C4HSF132 | C418S0268 | StP21 | 132.30 | 53.38 | 7.49 |
| C4HSF133 | C418S0269 | StP21 | 142.80 | 47.01 | 8.68 |
| C4HSF134 | C418S0270 | StP21 | 135.55 | 31.36 | 7.49 |
| C4HSF135 | C418S0273 | StP21 | 117.30 | 24.36 | 6.83 |
| C4HSF136 | C418S0274 | StP21 | 131.05 | 27.00 | 7.50 |
| C4HSF137 | C418S0276 | StP21 | 131.80 | 29.85 | 6.52 |
| C4HSF138 | C418S0278 | StP21 | 130.55 | 37.02 | 7.10 |
| C4HSF139 | C418S0280 | StP21 | 129.55 | 25.89 | 7.85 |
| C4HSF140 | C418S0281 | StP21 | 141.80 | 41.47 | 8.76 |
| C4HSF141 | C418S0282 | StP21 | 128.55 | 40.72 | 8.66 |
| C4HSF142 | C418S0283 | StP21 | 131.30 | 21.50 | 8.17 |
| C4HSF143 | C418S0285 | StP21 | 135.30 | 21.22 | 6.86 |
| C4HSF144 | C418S0289 | StP21 | 122.05 | 22.20 | 7.12 |
| C4HSF145 | C418S0291 | StP21 | 131.97 | 39.30 | 7.53 |
| C4HSF146 | C418S0298 | StP21 | 129.80 | 27.25 | 7.34 |
| C4HSF147 | C418S0301 | StP21 | 132.55 | 28.43 | 7.38 |
| C4HSF148 | C418S0302 | StP21 | 132.30 | 27.41 | 7.12 |
| C4HSF149 | C418S0303 | StP21 | 137.55 | 26.60 | 6.56 |
| C4HSF150 | C418S0304 | StP21 | 137.30 | 26.41 | 7.54 |
| C4HSF151 | C418S0305 | StP21 | 132.05 | 24.37 | 7.37 |
| C4HSF152 | C418S0308 | StP21 | 121.55 | 26.96 | 6.81 |
| C4HSF153 | C418S0309 | StP21 | 125.80 | 29.57 | 6.92 |
| C4HSF154 | C418S0315 | StP21 | 135.55 | 32.21 | 7.00 |
| C4HSF155 | C418S0321 | StP21 | 133.64 | 28.55 | 6.98 |
| C4HSF156 | C418S0325 | StP21 | 133.55 | 40.48 | 9.17 |
| C4HSF157 | C418S0327 | StP21 | 131.55 | 19.76 | 6.81 |
| C4HSF158 | C418S0330 | StP21 | 134.05 | 42.44 | 9.08 |
| C4HSF159 | C418S0336 | StP21 | 127.30 | 44.27 | 7.73 |
| C4HSF160 | C418S0337 | StP21 | 127.55 | 37.10 | 8.41 |
| C4HSF161 | C418S0340 | StP21 | 132.05 | 28.01 | 7.32 |
| C4HSF162 | C418S0343 | StP21 | 133.05 | 22.58 | 7.65 |
| C4HSF163 | C418S0344 | StP21 | 127.05 | 29.74 | 8.08 |
| C4HSF164 | C418S0350 | StP21 | 140.30 | 27.34 | 8.01 |
| C4HSF165 | C418S0351 | StP21 | 133.05 | 48.46 | 7.72 |
| C4HSF166 | C418S0352 | StP21 | 136.05 | 34.61 | 7.32 |
| C4HSF167 | C418S0353 | StP21 | 136.30 | 28.64 | 6.97 |
| C4HSF168 | C418S0355 | StP21 | 135.30 | 19.14 | 6.66 |
| C4HSF169 | C418S0356 | StP21 | 134.30 | 31.89 | 8.37 |
| C4HSF170 | C418S0357 | StP21 | 132.05 | 19.59 | 7.90 |
| C4HSF171 | C418S0358 | StP21 | 131.30 | 27.27 | 7.49 |
| C4HSF172 | C418S0360 | StP21 | 139.55 | 32.38 | 7.70 |
| C4HSF173 | C418S0362 | StP21 | 137.80 | 41.18 | 7.61 |
| C4HSF174 | C418S0364 | StP21 | 137.30 | 39.06 | 7.21 |
| C4HSF175 | C418S0365 | StP21 | 136.05 | 19.17 | 6.99 |
| C4HSF176 | C418S0367 | StP21 | 135.05 | 42.53 | 7.21 |
| C4HSF177 | C418S0368 | StP21 | 127.80 | NA | NA |
| C4HSF178 | C418S0369 | StP21 | 118.05 | 61.38 | 7.62 |
| C4HSF179 | C418S0382 | StP21 | 130.80 | 41.36 | 5.76 |
| C4HSF180 | C418S0385 | StP21 | 128.30 | 43.83 | 7.86 |
| C4HSF181 | C418S0386 | StP21 | 134.05 | 34.26 | 6.79 |
| C4HSF182 | C418S0390 | StP21 | 139.30 | 38.28 | 7.98 |
| C4HSF183 | C418S0391 | StP21 | 126.30 | 17.95 | 7.08 |
| C4HSF184 | C418S0392 | StP21 | 135.55 | 15.81 | 8.41 |
| C4HSF185 | C418S0394 | StP21 | 139.80 | 37.90 | 7.44 |
| C4HSF186 | C418S0396 | StP21 | 131.30 | 29.94 | 7.18 |
| C4HSF187 | C418S0399 | StP21 | 124.55 | 19.54 | 7.05 |
| C4HSF188 | C418S0401 | StP21 | 123.80 | 39.76 | 8.06 |
| C4HSF189 | C418S0406 | StP21 | 135.05 | 32.86 | 7.76 |
| C4HSF190 | C418S0410 | StP21 | 134.30 | 28.19 | 7.36 |
| C4HSF191 | C418S0411 | StP21 | 137.30 | 43.12 | 9.46 |
| C4HSF192 | C418S0412 | StP21 | 132.55 | 30.94 | 6.95 |
| C4HSF193 | C418S0413 | StP21 | 140.30 | 43.30 | 7.80 |
| C4HSF194 | C418S0414 | StP21 | 136.30 | 32.42 | 7.38 |
| C4HSF195 | C418S0420 | StP21 | 140.05 | 22.98 | 7.98 |
| C4HSF196 | C418S0421 | StP21 | 122.05 | 45.00 | 7.39 |
| C4HSF197 | C418S0422 | StP21 | 131.05 | 28.16 | 7.70 |
| C4HSF198 | C418S0423 | StP21 | 125.55 | 25.34 | 8.21 |
| C4HSF199 | C418S0426 | StP21 | 143.55 | 23.30 | 7.20 |
| C4HSF200 | C418S0427 | StP21 | 132.30 | 32.53 | 7.10 |
| C4HSF201 | C418S0428 | StP21 | 131.55 | 26.32 | 9.26 |
| C4HSF202 | C418S0429 | StP21 | 119.80 | NA | NA |
| C4HSF203 | C418S0430 | StP21 | 115.55 | 20.00 | 5.63 |
| C4HSF204 | C418S0455 | StP21 | 133.55 | 23.15 | 7.45 |
| C4HSF205 | C418S0458 | StP21 | 129.30 | 39.34 | 7.06 |
| C4HSF206 | C418S0463 | StP21 | 121.30 | 34.71 | 7.57 |
| C4HSF211 | C418S0489 | StP21 | 128.55 | 52.70 | 6.26 |
| C4HSF212 | C418S0491 | StP21 | 148.30 | NA | NA |
| C4HSF213 | C418S0492 | StP21 | 133.05 | 27.02 | 7.77 |
| C4HSF217 | C418S0497 | StP21 | 124.30 | 33.89 | 7.98 |
| C4HSF223 | C418S0572 | StP21 | 125.80 | 31.16 | 7.29 |
| C4HSF001 | C418S0213 | StP22 | 136.95 | 27.70 | 8.01 |
| C4HSF002 | C418S0228 | StP22 | 135.95 | 18.53 | 7.83 |
| C4HSF003 | C418S0252 | StP22 | 143.20 | 24.36 | 7.04 |
| C4HSF004 | C418S0263 | StP22 | 137.95 | 37.78 | 7.30 |
| C4HSF005 | C418S0284 | StP22 | 116.95 | 26.03 | 6.92 |
| C4HSF006 | C418S0286 | StP22 | 131.95 | 29.36 | 7.74 |
| C4HSF007 | C418S0425 | StP22 | 124.45 | 35.39 | 7.91 |
| C4HSF008 | C418S0469 | StP22 | 128.70 | 28.24 | 7.25 |
| C4HSF009 | C418S0499 | StP22 | 131.45 | 35.38 | 7.81 |
| C4HSF010 | C418S0565 | StP22 | 132.45 | 40.44 | 7.14 |
| C4HSF011 | C418S0619 | StP22 | 139.70 | 41.22 | 7.91 |
| C4HSF012 | C418S0003 | StP22 | 122.20 | 17.64 | 7.55 |
| C4HSF013 | C418S0004 | StP22 | 127.20 | 27.42 | 7.23 |
| C4HSF014 | C418S0006 | StP22 | 143.95 | 35.65 | 7.81 |
| C4HSF015 | C418S0007 | StP22 | 121.61 | 36.49 | 7.02 |
| C4HSF016 | C418S0008 | StP22 | 129.61 | 33.35 | 7.95 |
| C4HSF017 | C418S0009 | StP22 | 125.45 | 27.82 | 7.73 |
| C4HSF018 | C418S0010 | StP22 | 131.20 | 20.43 | 7.65 |
| C4HSF019 | C418S0014 | StP22 | 131.45 | 34.78 | 8.41 |
| C4HSF020 | C418S0015 | StP22 | 131.70 | 25.98 | 8.06 |
| C4HSF021 | C418S0017 | StP22 | 140.70 | 35.61 | 6.55 |
| C4HSF022 | C418S0020 | StP22 | 137.45 | 31.65 | 7.20 |
| C4HSF023 | C418S0021 | StP22 | 134.70 | 18.83 | 7.39 |
| C4HSF024 | C418S0022 | StP22 | 133.95 | 37.19 | 9.48 |
| C4HSF025 | C418S0023 | StP22 | 132.95 | 37.57 | 7.04 |
| C4HSF026 | C418S0024 | StP22 | 129.70 | 22.57 | 7.25 |
| C4HSF027 | C418S0025 | StP22 | 126.20 | 49.58 | 7.04 |
| C4HSF028 | C418S0026 | StP22 | 123.20 | 25.51 | 6.79 |
| C4HSF029 | C418S0028 | StP22 | 115.95 | 30.93 | 6.82 |
| C4HSF030 | C418S0031 | StP22 | 135.78 | 32.44 | 7.17 |
| C4HSF031 | C418S0032 | StP22 | 133.20 | 32.51 | 7.87 |
| C4HSF032 | C418S0034 | StP22 | 124.45 | 35.38 | 7.04 |
| C4HSF033 | C418S0035 | StP22 | 127.20 | 41.78 | 7.22 |
| C4HSF034 | C418S0051 | StP22 | 136.20 | 24.80 | 7.54 |
| C4HSF035 | C418S0054 | StP22 | 123.45 | 26.56 | 8.38 |
| C4HSF036 | C418S0056 | StP22 | 145.95 | 31.08 | 8.37 |
| C4HSF037 | C418S0061 | StP22 | 132.20 | 44.55 | 6.98 |
| C4HSF038 | C418S0062 | StP22 | 134.70 | 32.38 | 7.83 |
| C4HSF039 | C418S0063 | StP22 | 129.45 | 34.11 | 7.43 |
| C4HSF040 | C418S0068 | StP22 | 133.70 | 24.24 | 7.58 |
| C4HSF041 | C418S0079 | StP22 | 124.70 | 25.60 | 6.90 |
| C4HSF042 | C418S0084 | StP22 | 135.20 | 31.55 | 7.42 |
| C4HSF043 | C418S0086 | StP22 | 121.45 | 32.85 | 8.17 |
| C4HSF044 | C418S0087 | StP22 | 131.70 | 27.59 | 7.51 |
| C4HSF045 | C418S0089 | StP22 | 135.45 | 34.84 | 6.87 |
| C4HSF046 | C418S0092 | StP22 | 134.28 | 17.60 | 8.02 |
| C4HSF047 | C418S0093 | StP22 | 117.61 | 37.75 | 8.09 |
| C4HSF049 | C418S0097 | StP22 | 127.70 | 30.00 | 7.00 |
| C4HSF050 | C418S0098 | StP22 | 134.20 | 32.99 | 6.96 |
| C4HSF051 | C418S0100 | StP22 | 125.70 | 35.27 | 6.72 |
| C4HSF052 | C418S0103 | StP22 | 124.70 | 26.65 | 6.74 |
| C4HSF054 | C418S0118 | StP22 | 140.20 | 32.95 | 8.68 |
| C4HSF055 | C418S0119 | StP22 | 140.20 | 21.67 | 7.97 |
| C4HSF056 | C418S0120 | StP22 | 130.28 | 40.38 | 7.37 |
| C4HSF057 | C418S0121 | StP22 | 131.45 | 25.84 | 7.80 |
| C4HSF058 | C418S0122 | StP22 | 127.28 | 29.62 | 6.98 |
| C4HSF059 | C418S0124 | StP22 | 133.95 | 26.42 | 8.89 |
| C4HSF060 | C418S0125 | StP22 | 129.20 | 27.50 | 7.88 |
| C4HSF061 | C418S0126 | StP22 | 125.95 | 24.89 | 8.14 |
| C4HSF062 | C418S0128 | StP22 | 138.20 | 23.06 | 6.99 |
| C4HSF063 | C418S0129 | StP22 | 133.20 | 33.76 | 7.77 |
| C4HSF064 | C418S0131 | StP22 | 132.20 | 27.07 | 7.08 |
| C4HSF065 | C418S0133 | StP22 | 132.28 | 35.92 | 7.22 |
| C4HSF066 | C418S0134 | StP22 | 138.45 | 35.26 | 9.40 |
| C4HSF067 | C418S0135 | StP22 | 135.45 | 25.65 | 6.34 |
| C4HSF068 | C418S0137 | StP22 | 132.20 | 25.23 | 7.82 |
| C4HSF069 | C418S0138 | StP22 | 135.11 | 25.32 | 7.54 |
| C4HSF070 | C418S0139 | StP22 | 120.45 | 44.87 | 7.53 |
| C4HSF071 | C418S0140 | StP22 | 135.45 | 26.27 | 7.69 |
| C4HSF073 | C418S0143 | StP22 | 122.95 | 26.09 | 6.86 |
| C4HSF074 | C418S0145 | StP22 | 138.95 | 32.06 | 6.36 |
| C4HSF076 | C418S0149 | StP22 | 139.20 | 33.62 | 6.58 |
| C4HSF077 | C418S0150 | StP22 | 135.95 | 28.54 | 6.63 |
| C4HSF078 | C418S0151 | StP22 | 134.95 | 31.92 | 7.76 |
| C4HSF079 | C418S0152 | StP22 | 136.70 | 36.19 | 6.95 |
| C4HSF080 | C418S0153 | StP22 | 130.95 | 22.89 | 7.29 |
| C4HSF081 | C418S0154 | StP22 | 126.70 | 41.24 | 7.11 |
| C4HSF082 | C418S0156 | StP22 | 135.20 | 25.87 | 8.33 |
| C4HSF083 | C418S0157 | StP22 | 128.95 | 27.30 | 7.59 |
| C4HSF084 | C418S0161 | StP22 | 130.95 | 22.76 | 7.38 |
| C4HSF085 | C418S0163 | StP22 | 133.95 | 18.32 | 8.15 |
| C4HSF086 | C418S0164 | StP22 | 131.95 | 29.90 | 6.85 |
| C4HSF087 | C418S0165 | StP22 | 133.45 | 42.21 | 7.16 |
| C4HSF088 | C418S0166 | StP22 | 135.70 | 35.71 | 7.58 |
| C4HSF089 | C418S0167 | StP22 | 126.20 | 35.03 | 6.71 |
| C4HSF090 | C418S0168 | StP22 | 132.70 | 31.34 | 7.46 |
| C4HSF091 | C418S0169 | StP22 | 122.20 | 29.27 | 6.38 |
| C4HSF092 | C418S0171 | StP22 | 138.95 | 29.42 | 7.84 |
| C4HSF093 | C418S0172 | StP22 | 137.78 | 41.16 | 7.74 |
| C4HSF094 | C418S0177 | StP22 | 138.28 | 30.00 | 6.53 |
| C4HSF095 | C418S0178 | StP22 | 133.45 | 33.75 | 7.34 |
| C4HSF096 | C418S0180 | StP22 | 135.70 | 20.13 | 8.09 |
| C4HSF097 | C418S0193 | StP22 | 133.45 | 33.30 | 7.61 |
| C4HSF098 | C418S0196 | StP22 | 132.20 | 25.59 | 7.65 |
| C4HSF099 | C418S0197 | StP22 | 138.95 | 40.41 | 7.80 |
| C4HSF100 | C418S0199 | StP22 | 129.45 | 38.24 | 7.03 |
| C4HSF101 | C418S0201 | StP22 | 126.70 | 27.54 | 7.29 |
| C4HSF102 | C418S0202 | StP22 | 135.95 | 25.80 | 8.08 |
| C4HSF103 | C418S0206 | StP22 | 135.20 | 40.61 | 8.37 |
| C4HSF104 | C418S0207 | StP22 | 133.95 | 25.47 | 7.85 |
| C4HSF105 | C418S0208 | StP22 | 135.20 | 38.86 | 8.67 |
| C4HSF106 | C418S0209 | StP22 | 126.70 | 36.46 | 8.13 |
| C4HSF107 | C418S0210 | StP22 | 128.70 | 31.69 | 6.83 |
| C4HSF108 | C418S0215 | StP22 | 125.70 | 30.24 | 6.74 |
| C4HSF109 | C418S0217 | StP22 | 129.70 | 34.97 | 5.95 |
| C4HSF110 | C418S0219 | StP22 | 129.70 | 20.06 | 7.39 |
| C4HSF111 | C418S0220 | StP22 | 133.45 | 35.39 | 7.34 |
| C4HSF112 | C418S0225 | StP22 | 122.45 | 31.59 | 6.39 |
| C4HSF113 | C418S0226 | StP22 | 123.45 | 30.20 | 6.81 |
| C4HSF114 | C418S0230 | StP22 | 139.28 | 41.67 | 7.23 |
| C4HSF115 | C418S0232 | StP22 | 135.70 | 22.16 | 6.92 |
| C4HSF116 | C418S0233 | StP22 | 140.20 | 43.13 | 7.22 |
| C4HSF117 | C418S0234 | StP22 | 136.95 | 30.43 | 7.63 |
| C4HSF118 | C418S0235 | StP22 | 121.95 | 31.86 | 7.50 |
| C4HSF119 | C418S0236 | StP22 | 134.20 | 30.75 | 8.08 |
| C4HSF120 | C418S0237 | StP22 | 131.45 | 28.50 | 6.24 |
| C4HSF121 | C418S0240 | StP22 | 132.95 | 29.11 | 6.86 |
| C4HSF122 | C418S0242 | StP22 | 145.70 | 27.74 | 7.37 |
| C4HSF123 | C418S0243 | StP22 | 129.78 | 27.39 | 7.11 |
| C4HSF124 | C418S0244 | StP22 | 137.20 | 42.49 | 8.49 |
| C4HSF125 | C418S0245 | StP22 | 132.45 | 28.35 | 8.11 |
| C4HSF126 | C418S0253 | StP22 | 138.95 | 33.06 | 7.22 |
| C4HSF127 | C418S0254 | StP22 | 136.95 | 33.13 | 26.93 |
| C4HSF128 | C418S0259 | StP22 | 143.70 | 32.68 | 8.81 |
| C4HSF129 | C418S0260 | StP22 | 133.95 | 36.96 | 6.99 |
| C4HSF130 | C418S0264 | StP22 | 130.45 | 32.25 | 7.69 |
| C4HSF131 | C418S0267 | StP22 | 128.70 | 32.84 | 7.29 |
| C4HSF132 | C418S0268 | StP22 | 134.20 | 37.11 | 7.69 |
| C4HSF133 | C418S0269 | StP22 | 133.20 | 30.52 | 8.23 |
| C4HSF134 | C418S0270 | StP22 | 135.70 | 39.89 | 7.81 |
| C4HSF135 | C418S0273 | StP22 | 134.20 | 37.11 | 7.64 |
| C4HSF136 | C418S0274 | StP22 | 130.70 | 16.81 | 7.41 |
| C4HSF137 | C418S0276 | StP22 | 127.95 | 29.20 | 6.74 |
| C4HSF138 | C418S0278 | StP22 | 137.20 | 29.69 | 7.61 |
| C4HSF139 | C418S0280 | StP22 | 133.45 | 17.39 | 7.45 |
| C4HSF140 | C418S0281 | StP22 | 129.45 | 24.51 | 7.08 |
| C4HSF141 | C418S0282 | StP22 | 127.95 | 27.31 | 8.42 |
| C4HSF142 | C418S0283 | StP22 | 128.70 | 37.68 | 6.54 |
| C4HSF143 | C418S0285 | StP22 | 130.20 | 26.56 | 7.20 |
| C4HSF144 | C418S0289 | StP22 | 122.45 | 19.68 | 7.36 |
| C4HSF145 | C418S0291 | StP22 | 130.78 | 26.33 | 8.19 |
| C4HSF146 | C418S0298 | StP22 | 136.95 | 43.14 | 7.87 |
| C4HSF147 | C418S0301 | StP22 | 131.45 | 15.32 | 7.85 |
| C4HSF148 | C418S0302 | StP22 | 128.95 | 32.98 | 8.16 |
| C4HSF149 | C418S0303 | StP22 | 131.20 | 40.06 | 7.48 |
| C4HSF150 | C418S0304 | StP22 | 136.95 | 36.72 | 7.00 |
| C4HSF151 | C418S0305 | StP22 | 127.95 | 41.07 | 7.24 |
| C4HSF152 | C418S0308 | StP22 | 122.45 | 29.20 | 7.38 |
| C4HSF153 | C418S0309 | StP22 | 122.45 | 33.12 | 6.31 |
| C4HSF154 | C418S0315 | StP22 | 126.20 | 23.72 | 6.34 |
| C4HSF155 | C418S0321 | StP22 | 133.95 | 25.52 | 6.66 |
| C4HSF156 | C418S0325 | StP22 | 147.20 | 22.09 | 7.36 |
| C4HSF157 | C418S0327 | StP22 | 130.95 | 34.49 | 8.03 |
| C4HSF158 | C418S0330 | StP22 | 139.20 | 24.02 | 8.31 |
| C4HSF159 | C418S0336 | StP22 | 141.20 | 27.27 | 6.96 |
| C4HSF160 | C418S0337 | StP22 | 143.45 | 24.59 | 7.73 |
| C4HSF161 | C418S0340 | StP22 | 127.95 | 25.39 | 6.39 |
| C4HSF162 | C418S0343 | StP22 | 137.20 | 34.47 | 7.64 |
| C4HSF163 | C418S0344 | StP22 | 122.20 | 37.88 | 9.00 |
| C4HSF164 | C418S0350 | StP22 | 126.20 | 21.56 | 6.72 |
| C4HSF165 | C418S0351 | StP22 | 123.20 | 34.10 | 6.28 |
| C4HSF166 | C418S0352 | StP22 | 133.45 | 37.08 | 6.47 |
| C4HSF167 | C418S0353 | StP22 | 123.70 | 32.34 | 6.81 |
| C4HSF168 | C418S0355 | StP22 | 133.95 | 27.33 | 6.87 |
| C4HSF169 | C418S0356 | StP22 | 132.45 | 30.17 | 6.74 |
| C4HSF170 | C418S0357 | StP22 | 138.20 | 32.75 | 7.01 |
| C4HSF171 | C418S0358 | StP22 | 132.45 | 33.14 | 6.64 |
| C4HSF172 | C418S0360 | StP22 | 137.70 | 30.28 | 6.80 |
| C4HSF173 | C418S0362 | StP22 | 140.95 | 32.67 | 8.01 |
| C4HSF174 | C418S0364 | StP22 | 129.70 | 28.45 | 8.42 |
| C4HSF175 | C418S0365 | StP22 | 133.70 | 27.61 | 7.71 |
| C4HSF176 | C418S0367 | StP22 | 145.45 | 26.72 | 7.51 |
| C4HSF177 | C418S0368 | StP22 | 137.45 | 27.21 | 7.29 |
| C4HSF178 | C418S0369 | StP22 | 131.45 | 21.58 | 8.40 |
| C4HSF179 | C418S0382 | StP22 | 129.45 | 29.75 | 7.14 |
| C4HSF180 | C418S0385 | StP22 | 129.95 | 27.20 | 16.14 |
| C4HSF181 | C418S0386 | StP22 | 131.45 | 36.13 | 6.98 |
| C4HSF182 | C418S0390 | StP22 | 139.20 | 49.03 | 7.70 |
| C4HSF183 | C418S0391 | StP22 | 135.20 | 33.79 | 8.13 |
| C4HSF184 | C418S0392 | StP22 | 133.20 | 55.80 | 7.59 |
| C4HSF185 | C418S0394 | StP22 | 139.45 | 21.39 | 7.75 |
| C4HSF186 | C418S0396 | StP22 | 130.95 | 34.54 | 6.68 |
| C4HSF187 | C418S0399 | StP22 | 119.20 | 19.43 | 7.88 |
| C4HSF188 | C418S0401 | StP22 | 132.45 | 27.31 | 8.20 |
| C4HSF189 | C418S0406 | StP22 | 138.70 | 34.53 | 7.32 |
| C4HSF190 | C418S0410 | StP22 | 135.70 | 35.68 | 7.29 |
| C4HSF191 | C418S0411 | StP22 | 139.45 | 29.17 | 8.47 |
| C4HSF192 | C418S0412 | StP22 | 137.45 | 43.65 | 7.16 |
| C4HSF193 | C418S0413 | StP22 | 147.20 | 25.70 | 7.42 |
| C4HSF194 | C418S0414 | StP22 | 143.20 | 33.49 | 8.16 |
| C4HSF195 | C418S0420 | StP22 | 139.95 | 26.25 | 7.49 |
| C4HSF196 | C418S0421 | StP22 | 138.70 | 33.34 | 8.04 |
| C4HSF197 | C418S0422 | StP22 | 130.45 | 35.03 | 7.21 |
| C4HSF198 | C418S0423 | StP22 | 123.70 | 30.31 | 7.67 |
| C4HSF199 | C418S0426 | StP22 | 133.20 | 26.19 | 6.65 |
| C4HSF200 | C418S0427 | StP22 | 128.11 | 28.97 | 6.61 |
| C4HSF201 | C418S0428 | StP22 | 115.45 | 31.17 | 7.06 |
| C4HSF202 | C418S0429 | StP22 | 128.70 | 18.99 | 6.67 |
| C4HSF203 | C418S0430 | StP22 | 116.20 | 29.60 | 6.18 |
| C4HSF204 | C418S0455 | StP22 | 122.45 | 38.42 | 7.13 |
| C4HSF205 | C418S0458 | StP22 | 125.70 | 42.80 | 6.28 |
| C4HSF206 | C418S0463 | StP22 | 133.11 | 42.13 | 7.05 |
| C4HSF211 | C418S0489 | StP22 | 120.95 | 34.32 | 7.17 |
| C4HSF212 | C418S0491 | StP22 | 120.20 | 25.83 | 6.59 |
| C4HSF213 | C418S0492 | StP22 | 125.95 | 30.53 | 7.07 |
| C4HSF217 | C418S0497 | StP22 | 130.95 | 30.43 | 6.40 |
| C4HSF223 | C418S0572 | StP22 | 118.78 | 31.91 | 6.36 |
| C4HSF001 | C418S0213 | StP23 | NA | 30.18 | 8.75 |
| C4HSF002 | C418S0228 | StP23 | NA | 30.24 | 7.92 |
| C4HSF003 | C418S0252 | StP23 | NA | 34.91 | 6.88 |
| C4HSF004 | C418S0263 | StP23 | NA | 37.81 | 7.90 |
| C4HSF005 | C418S0284 | StP23 | NA | 23.02 | 6.86 |
| C4HSF006 | C418S0286 | StP23 | NA | 30.08 | 7.37 |
| C4HSF007 | C418S0425 | StP23 | NA | 28.73 | 8.41 |
| C4HSF008 | C418S0469 | StP23 | NA | 28.99 | 7.56 |
| C4HSF009 | C418S0499 | StP23 | NA | 32.10 | 8.00 |
| C4HSF010 | C418S0565 | StP23 | NA | 31.13 | 7.67 |
| C4HSF011 | C418S0619 | StP23 | NA | 40.63 | 7.50 |
| C4HSF012 | C418S0003 | StP23 | 120.49 | 33.06 | 6.96 |
| C4HSF013 | C418S0004 | StP23 | NA | 21.81 | 7.60 |
| C4HSF014 | C418S0006 | StP23 | NA | 27.24 | 8.08 |
| C4HSF015 | C418S0007 | StP23 | 122.99 | 25.96 | 7.17 |
| C4HSF016 | C418S0008 | StP23 | NA | 33.49 | 8.12 |
| C4HSF017 | C418S0009 | StP23 | NA | 30.04 | 7.60 |
| C4HSF018 | C418S0010 | StP23 | NA | 26.28 | 8.08 |
| C4HSF019 | C418S0014 | StP23 | NA | 32.34 | 8.34 |
| C4HSF020 | C418S0015 | StP23 | NA | 30.87 | 8.69 |
| C4HSF021 | C418S0017 | StP23 | NA | 30.73 | 7.01 |
| C4HSF022 | C418S0020 | StP23 | 135.83 | 20.00 | 7.61 |
| C4HSF023 | C418S0021 | StP23 | NA | 18.93 | 7.75 |
| C4HSF024 | C418S0022 | StP23 | 124.83 | 37.77 | 10.56 |
| C4HSF025 | C418S0023 | StP23 | 139.83 | 28.85 | 7.42 |
| C4HSF026 | C418S0024 | StP23 | 125.83 | 23.55 | 7.28 |
| C4HSF027 | C418S0025 | StP23 | NA | 32.88 | 6.84 |
| C4HSF028 | C418S0026 | StP23 | NA | 22.85 | 6.64 |
| C4HSF029 | C418S0028 | StP23 | 121.49 | 67.40 | 8.05 |
| C4HSF030 | C418S0031 | StP23 | NA | 24.14 | 8.33 |
| C4HSF031 | C418S0032 | StP23 | NA | 29.02 | 8.41 |
| C4HSF032 | C418S0034 | StP23 | NA | 27.65 | 7.27 |
| C4HSF033 | C418S0035 | StP23 | 132.83 | 35.00 | 7.65 |
| C4HSF034 | C418S0051 | StP23 | NA | 25.25 | 7.87 |
| C4HSF035 | C418S0054 | StP23 | NA | 25.38 | 8.38 |
| C4HSF036 | C418S0056 | StP23 | NA | 27.69 | 8.59 |
| C4HSF037 | C418S0061 | StP23 | 137.83 | 37.21 | 7.33 |
| C4HSF038 | C418S0062 | StP23 | NA | 30.36 | 7.70 |
| C4HSF039 | C418S0063 | StP23 | NA | 34.20 | 7.39 |
| C4HSF040 | C418S0068 | StP23 | NA | 40.01 | 7.50 |
| C4HSF041 | C418S0079 | StP23 | NA | 34.70 | 6.89 |
| C4HSF042 | C418S0084 | StP23 | 138.83 | 23.84 | 7.89 |
| C4HSF043 | C418S0086 | StP23 | NA | 30.00 | 8.14 |
| C4HSF044 | C418S0087 | StP23 | NA | 31.03 | 7.35 |
| C4HSF045 | C418S0089 | StP23 | NA | 28.69 | 7.52 |
| C4HSF046 | C418S0092 | StP23 | NA | 34.68 | 7.76 |
| C4HSF047 | C418S0093 | StP23 | NA | 29.07 | 8.41 |
| C4HSF049 | C418S0097 | StP23 | NA | 30.96 | 6.87 |
| C4HSF050 | C418S0098 | StP23 | NA | 36.13 | 7.52 |
| C4HSF051 | C418S0100 | StP23 | 136.83 | 29.01 | 7.12 |
| C4HSF052 | C418S0103 | StP23 | NA | 31.94 | 7.02 |
| C4HSF054 | C418S0118 | StP23 | NA | 44.66 | 9.73 |
| C4HSF055 | C418S0119 | StP23 | 127.16 | 33.43 | 8.09 |
| C4HSF056 | C418S0120 | StP23 | 128.49 | 26.37 | 7.87 |
| C4HSF057 | C418S0121 | StP23 | NA | 27.15 | 7.64 |
| C4HSF058 | C418S0122 | StP23 | NA | 33.19 | 7.08 |
| C4HSF059 | C418S0124 | StP23 | NA | 37.46 | 7.88 |
| C4HSF060 | C418S0125 | StP23 | NA | 28.93 | 7.11 |
| C4HSF061 | C418S0126 | StP23 | NA | 34.80 | 7.10 |
| C4HSF062 | C418S0128 | StP23 | 130.83 | 32.24 | 6.99 |
| C4HSF063 | C418S0129 | StP23 | NA | 33.88 | 7.93 |
| C4HSF064 | C418S0131 | StP23 | NA | 29.51 | 6.60 |
| C4HSF065 | C418S0133 | StP23 | NA | 32.48 | 7.41 |
| C4HSF066 | C418S0134 | StP23 | NA | 32.33 | 8.07 |
| C4HSF067 | C418S0135 | StP23 | NA | 38.23 | 7.08 |
| C4HSF068 | C418S0137 | StP23 | NA | 34.63 | 7.97 |
| C4HSF069 | C418S0138 | StP23 | 133.49 | 26.08 | 7.39 |
| C4HSF070 | C418S0139 | StP23 | NA | 30.86 | 7.20 |
| C4HSF071 | C418S0140 | StP23 | NA | 37.94 | 7.41 |
| C4HSF073 | C418S0143 | StP23 | 132.83 | 42.61 | 7.10 |
| C4HSF074 | C418S0145 | StP23 | 140.49 | 27.51 | 7.00 |
| C4HSF076 | C418S0149 | StP23 | NA | 29.79 | 7.23 |
| C4HSF077 | C418S0150 | StP23 | NA | 42.50 | 7.49 |
| C4HSF078 | C418S0151 | StP23 | 135.49 | 31.13 | 7.08 |
| C4HSF079 | C418S0152 | StP23 | NA | 34.87 | 7.70 |
| C4HSF080 | C418S0153 | StP23 | NA | 34.36 | 7.08 |
| C4HSF081 | C418S0154 | StP23 | 139.16 | 34.13 | 7.42 |
| C4HSF082 | C418S0156 | StP23 | NA | 31.45 | 8.59 |
| C4HSF083 | C418S0157 | StP23 | NA | 27.71 | 8.39 |
| C4HSF084 | C418S0161 | StP23 | NA | 34.08 | 7.99 |
| C4HSF085 | C418S0163 | StP23 | NA | 23.63 | 8.06 |
| C4HSF086 | C418S0164 | StP23 | NA | 26.25 | 6.50 |
| C4HSF087 | C418S0165 | StP23 | NA | 31.85 | 7.52 |
| C4HSF088 | C418S0166 | StP23 | NA | 24.41 | 8.78 |
| C4HSF089 | C418S0167 | StP23 | NA | 21.69 | 6.29 |
| C4HSF090 | C418S0168 | StP23 | 142.16 | 19.80 | 7.66 |
| C4HSF091 | C418S0169 | StP23 | NA | 20.67 | 7.03 |
| C4HSF092 | C418S0171 | StP23 | 131.16 | 34.51 | 7.70 |
| C4HSF093 | C418S0172 | StP23 | NA | 31.48 | 7.61 |
| C4HSF094 | C418S0177 | StP23 | NA | 24.93 | 6.78 |
| C4HSF095 | C418S0178 | StP23 | NA | 34.20 | 7.39 |
| C4HSF096 | C418S0180 | StP23 | 127.16 | 18.18 | 7.63 |
| C4HSF097 | C418S0193 | StP23 | NA | 37.40 | 7.83 |
| C4HSF098 | C418S0196 | StP23 | NA | 25.88 | 7.05 |
| C4HSF099 | C418S0197 | StP23 | NA | 30.61 | 6.95 |
| C4HSF100 | C418S0199 | StP23 | 111.99 | 31.28 | 7.50 |
| C4HSF101 | C418S0201 | StP23 | NA | 24.58 | 7.46 |
| C4HSF102 | C418S0202 | StP23 | 121.16 | 30.49 | 7.44 |
| C4HSF103 | C418S0206 | StP23 | NA | 37.06 | 8.83 |
| C4HSF104 | C418S0207 | StP23 | NA | 31.35 | 7.81 |
| C4HSF105 | C418S0208 | StP23 | NA | 28.18 | 7.54 |
| C4HSF106 | C418S0209 | StP23 | NA | 40.86 | 8.26 |
| C4HSF107 | C418S0210 | StP23 | NA | 30.69 | 7.00 |
| C4HSF108 | C418S0215 | StP23 | NA | 33.62 | 6.99 |
| C4HSF109 | C418S0217 | StP23 | NA | 23.50 | 6.24 |
| C4HSF110 | C418S0219 | StP23 | NA | 29.08 | 7.04 |
| C4HSF111 | C418S0220 | StP23 | NA | 30.95 | 7.55 |
| C4HSF112 | C418S0225 | StP23 | NA | 30.92 | 6.63 |
| C4HSF113 | C418S0226 | StP23 | 142.16 | 25.18 | 7.03 |
| C4HSF114 | C418S0230 | StP23 | NA | 25.12 | 7.69 |
| C4HSF115 | C418S0232 | StP23 | NA | 24.29 | 7.28 |
| C4HSF116 | C418S0233 | StP23 | NA | 33.54 | 7.78 |
| C4HSF117 | C418S0234 | StP23 | NA | 21.36 | 7.92 |
| C4HSF118 | C418S0235 | StP23 | NA | 26.84 | 7.36 |
| C4HSF119 | C418S0236 | StP23 | NA | 32.99 | 7.58 |
| C4HSF120 | C418S0237 | StP23 | 136.83 | 31.56 | 7.28 |
| C4HSF121 | C418S0240 | StP23 | NA | 31.11 | 7.29 |
| C4HSF122 | C418S0242 | StP23 | NA | 30.60 | 7.52 |
| C4HSF123 | C418S0243 | StP23 | NA | 30.86 | 7.37 |
| C4HSF124 | C418S0244 | StP23 | NA | 36.26 | 8.33 |
| C4HSF125 | C418S0245 | StP23 | NA | 25.47 | 7.69 |
| C4HSF126 | C418S0253 | StP23 | NA | 26.93 | 7.45 |
| C4HSF127 | C418S0254 | StP23 | NA | 28.99 | 7.66 |
| C4HSF128 | C418S0259 | StP23 | NA | 31.59 | 7.74 |
| C4HSF129 | C418S0260 | StP23 | NA | 30.01 | 7.24 |
| C4HSF130 | C418S0264 | StP23 | NA | 34.08 | 8.58 |
| C4HSF131 | C418S0267 | StP23 | NA | 26.81 | 7.53 |
| C4HSF132 | C418S0268 | StP23 | 127.49 | 31.93 | 8.27 |
| C4HSF133 | C418S0269 | StP23 | NA | 36.75 | 7.20 |
| C4HSF134 | C418S0270 | StP23 | NA | 28.26 | 7.94 |
| C4HSF135 | C418S0273 | StP23 | NA | 35.80 | 7.51 |
| C4HSF136 | C418S0274 | StP23 | NA | 28.08 | 7.50 |
| C4HSF137 | C418S0276 | StP23 | NA | 29.58 | 6.77 |
| C4HSF138 | C418S0278 | StP23 | NA | 39.90 | 7.51 |
| C4HSF139 | C418S0280 | StP23 | NA | 29.19 | 6.93 |
| C4HSF140 | C418S0281 | StP23 | 139.83 | 23.30 | 6.90 |
| C4HSF141 | C418S0282 | StP23 | NA | 32.66 | 7.94 |
| C4HSF142 | C418S0283 | StP23 | NA | 33.82 | 6.55 |
| C4HSF143 | C418S0285 | StP23 | NA | 26.84 | 8.28 |
| C4HSF144 | C418S0289 | StP23 | NA | 30.66 | 8.09 |
| C4HSF145 | C418S0291 | StP23 | 140.49 | 41.32 | 8.73 |
| C4HSF146 | C418S0298 | StP23 | 142.49 | 28.03 | 8.00 |
| C4HSF147 | C418S0301 | StP23 | NA | 35.21 | 7.10 |
| C4HSF148 | C418S0302 | StP23 | NA | 30.87 | 7.59 |
| C4HSF149 | C418S0303 | StP23 | 138.99 | 30.01 | 7.39 |
| C4HSF150 | C418S0304 | StP23 | NA | 29.95 | 7.02 |
| C4HSF151 | C418S0305 | StP23 | NA | 31.06 | 7.65 |
| C4HSF152 | C418S0308 | StP23 | NA | 34.07 | 6.99 |
| C4HSF153 | C418S0309 | StP23 | NA | 31.38 | 7.79 |
| C4HSF154 | C418S0315 | StP23 | NA | 29.53 | 7.25 |
| C4HSF155 | C418S0321 | StP23 | NA | 24.42 | 6.38 |
| C4HSF156 | C418S0325 | StP23 | 132.16 | 29.79 | 7.64 |
| C4HSF157 | C418S0327 | StP23 | NA | 36.73 | 9.09 |
| C4HSF158 | C418S0330 | StP23 | 132.49 | 30.79 | 8.02 |
| C4HSF159 | C418S0336 | StP23 | NA | 35.60 | 7.64 |
| C4HSF160 | C418S0337 | StP23 | NA | 25.53 | 7.98 |
| C4HSF161 | C418S0340 | StP23 | NA | 21.71 | 7.50 |
| C4HSF162 | C418S0343 | StP23 | 130.83 | 31.67 | 8.27 |
| C4HSF163 | C418S0344 | StP23 | NA | 37.34 | 8.80 |
| C4HSF164 | C418S0350 | StP23 | NA | 24.32 | 6.87 |
| C4HSF165 | C418S0351 | StP23 | NA | 31.50 | 6.99 |
| C4HSF166 | C418S0352 | StP23 | NA | 26.33 | 6.96 |
| C4HSF167 | C418S0353 | StP23 | 130.99 | 23.38 | 7.69 |
| C4HSF168 | C418S0355 | StP23 | NA | 29.32 | 6.64 |
| C4HSF169 | C418S0356 | StP23 | NA | 32.60 | 7.01 |
| C4HSF170 | C418S0357 | StP23 | NA | 29.41 | 7.33 |
| C4HSF171 | C418S0358 | StP23 | NA | 28.17 | 6.21 |
| C4HSF172 | C418S0360 | StP23 | NA | 32.36 | 6.91 |
| C4HSF173 | C418S0362 | StP23 | NA | 27.22 | 7.82 |
| C4HSF174 | C418S0364 | StP23 | 123.49 | 33.06 | 8.56 |
| C4HSF175 | C418S0365 | StP23 | NA | 31.09 | 8.32 |
| C4HSF176 | C418S0367 | StP23 | NA | 27.35 | 7.34 |
| C4HSF177 | C418S0368 | StP23 | 137.49 | 42.86 | 7.28 |
| C4HSF178 | C418S0369 | StP23 | NA | 38.58 | 8.07 |
| C4HSF179 | C418S0382 | StP23 | NA | 34.13 | 7.41 |
| C4HSF180 | C418S0385 | StP23 | 110.83 | 33.00 | 7.06 |
| C4HSF181 | C418S0386 | StP23 | NA | 29.05 | 6.92 |
| C4HSF182 | C418S0390 | StP23 | NA | 35.01 | 7.83 |
| C4HSF183 | C418S0391 | StP23 | NA | 40.40 | 7.57 |
| C4HSF184 | C418S0392 | StP23 | NA | 29.79 | 7.91 |
| C4HSF185 | C418S0394 | StP23 | 130.49 | 24.51 | 7.66 |
| C4HSF186 | C418S0396 | StP23 | NA | 23.96 | 7.32 |
| C4HSF187 | C418S0399 | StP23 | NA | 37.73 | 7.61 |
| C4HSF188 | C418S0401 | StP23 | NA | 44.94 | 8.53 |
| C4HSF189 | C418S0406 | StP23 | NA | 30.18 | 7.51 |
| C4HSF190 | C418S0410 | StP23 | NA | 29.92 | 7.66 |
| C4HSF191 | C418S0411 | StP23 | 131.83 | 44.79 | 8.20 |
| C4HSF192 | C418S0412 | StP23 | 139.83 | 35.12 | 7.23 |
| C4HSF193 | C418S0413 | StP23 | NA | 28.63 | 8.31 |
| C4HSF194 | C418S0414 | StP23 | NA | 31.14 | 7.43 |
| C4HSF195 | C418S0420 | StP23 | NA | 29.31 | 7.97 |
| C4HSF196 | C418S0421 | StP23 | NA | 25.58 | 8.40 |
| C4HSF197 | C418S0422 | StP23 | NA | 27.39 | 7.46 |
| C4HSF198 | C418S0423 | StP23 | NA | 39.76 | 7.42 |
| C4HSF199 | C418S0426 | StP23 | 133.49 | 32.42 | 7.60 |
| C4HSF200 | C418S0427 | StP23 | NA | 22.09 | 6.86 |
| C4HSF201 | C418S0428 | StP23 | NA | 33.75 | 7.06 |
| C4HSF202 | C418S0429 | StP23 | NA | 30.52 | 6.36 |
| C4HSF203 | C418S0430 | StP23 | NA | 29.85 | 6.84 |
| C4HSF204 | C418S0455 | StP23 | NA | 39.13 | 7.31 |
| C4HSF205 | C418S0458 | StP23 | NA | 28.38 | 6.71 |
| C4HSF206 | C418S0463 | StP23 | NA | 32.03 | 6.84 |
| C4HSF211 | C418S0489 | StP23 | NA | 35.52 | 6.69 |
| C4HSF212 | C418S0491 | StP23 | NA | 29.87 | 6.64 |
| C4HSF213 | C418S0492 | StP23 | NA | 32.25 | 7.00 |
| C4HSF217 | C418S0497 | StP23 | NA | 32.10 | 6.82 |
| C4HSF223 | C418S0572 | StP23 | NA | 34.24 | 6.49 |
