# Supplementary material for: Assessment of genomic prediction and genetic gain in multi‑population half-sib families in the perennial grass crop intermediate wheatgrass
Source: PLoS One. 2026 Sep 2;21(9):e0357452. doi: 10.1371/journal.pone.0357452 (PMC13537581; doi:10.1371/journal.pone.0357452)
Supplement: Supplementary file 3 [file pone.0357452.s003.docx]

**S3 Table:** Cross-environment genomic predictions in the half-sib families for the traits plant height, grain yield, and seed size measured as thousand kernel weight (TKW). Multiple environment predictions were made with the multi-environment (MDe) model with training populations shows in rows and predicted populations in columns.

|  | **Lam21** | | | **Lam22** | | | **StP21** | | | **StP22** | | | **StP23** | | |
| --- | --- | --- | --- | --- | --- | --- | --- | --- | --- | --- | --- | --- | --- | --- | --- |
|  | **Height** | **Yield** | **TKW** | **Height** | **Yield** | **TKW** | **Height** | **Yield** | **TKW** | **Height** | **Yield** | **TKW** | **Height** | **Yield** | **TKW** |
| Y1 both sites | NA | NA | NA | 0.11 (0.14) | 0.08 (0.05) | 0.02 (0.06) | NA | NA | NA | 0.08 (0.05) | -0.05 (0.05) | 0.04 (0.06) | 0.10 (0.12) | 0.01 (0.06) | 0.11 (0.07) |
| Y2 both sites | 0.14 (0.10) | 0.01 (0.05) | 0.03 (0.06) | NA | NA | NA | 0.04 (0.06) | 0.02 (0.05) | 0.03 (0.07) | NA | NA | NA | 0.08 (0.12) | 0.05 (0.06) | 0.16 (0.12) |
| Lam all years | NA | NA | NA | NA | NA | NA | 0.01 (0.05) | 0.05 (0.05) | 0.03 (0.05) | 0.02 (0.04) | 0.01 (0.05) | -0.03 (0.06) | 0.02 (0.08) | -0.02 (0.06) | 0.01 (0.06) |
| StP all years | 0.02 (0.04) | -0.03 (0.06) | -0.01 (0.04) | -0.03 (0.05) | 0.05 (0.06) | -0.01 (0.06) | NA | NA | NA | NA | NA | NA | NA | NA | NA |
