# Supplementary material for: Assessment of genomic prediction and genetic gain in multi‑population half-sib families in the perennial grass crop intermediate wheatgrass
Source: PLoS One. 2026 Sep 2;21(9):e0357452. doi: 10.1371/journal.pone.0357452 (PMC13537581; doi:10.1371/journal.pone.0357452)
Supplement: Supplementary file 4 [file pone.0357452.s004.docx]

**S4 Table:** Weather data of Lamberton, MN and St. Paul, MN between 2020 and 2023. Information obtained from https://www.weather.gov; accessed 03-17-25

| Monthly Mean Precipitation, Lamberton | | | | | | | | | | | | |
| --- | --- | --- | --- | --- | --- | --- | --- | --- | --- | --- | --- | --- |
| Year | Jan | Feb | Mar | Apr | May | Jun | Jul | Aug | Sep | Oct | Nov | Dec |
| 2000 | 0.01 | 0.05 | 0.02 | 0.04 | 0.21 | 0.08 | 0.15 | 0.12 | 0.02 | 0.07 | 0.08 | 0.03 |
| 2001 | 0.04 | 0.04 | 0.03 | 0.29 | 0.07 | 0.07 | 0.21 | 0.05 | 0.12 | 0.02 | 0.14 | 0.01 |
| 2002 | 0 | 0.01 | 0.04 | 0.06 | 0.07 | 0.13 | 0.11 | 0.17 | 0.03 | 0.11 | 0 | 0.01 |
| 2003 | 0.01 | 0.02 | 0.03 | 0.06 | 0.09 | 0.18 | 0.05 | 0.04 | 0.12 | 0 | 0.02 | 0.02 |
| 2004 | 0.01 | 0.02 | 0.04 | 0.04 | 0.22 | 0.12 | 0.12 | 0.16 | 0.19 | 0.02 | 0.03 | 0 |
| 2005 | 0.02 | 0.02 | 0.03 | 0.12 | 0.19 | 0.13 | 0.1 | 0.09 | 0.33 | 0.05 | 0.07 | 0.04 |
| 2006 | 0.01 | 0.01 | 0.07 | 0.12 | 0.08 | 0.31 | 0.06 | 0.13 | 0.09 | 0.01 | 0.02 | 0.06 |
| 2007 | 0.01 | 0.03 | 0.06 | 0.1 | 0.08 | 0.12 | 0.03 | 0.18 | 0.06 | 0.2 | 0 | 0.01 |
| 2008 | 0 | 0 | 0.04 | 0.1 | 0.1 | 0.12 | 0.11 | 0.02 | 0.07 | 0.14 | 0.03 | 0.03 |
| 2009 | 0.01 | 0.02 | 0.04 | 0.05 | 0.05 | 0.11 | 0.05 | 0.11 | 0.09 | 0.17 | 0.01 | NA |
| 2010 | 0.03 | 0.03 | 0.04 | 0.08 | 0.06 | 0.21 | 0.12 | 0.15 | 0.35 | 0.06 | 0.03 | 0.05 |
| 2011 | 0.03 | 0.06 | 0.05 | 0.09 | 0.16 | 0.28 | 0.12 | 0.02 | NA | 0.02 | 0 | 0.02 |
| 2012 | 0.02 | 0.06 | 0.04 | 0.09 | 0.32 | 0.04 | 0.02 | 0.1 | 0.04 | 0.03 | 0.03 | 0.03 |
| 2013 | 0.01 | 0.02 | 0.03 | 0.11 | 0.14 | 0.18 | 0.01 | 0.06 | 0.06 | 0.1 | 0.02 | 0.03 |
| 2014 | 0.02 | 0.02 | 0.03 | 0.11 | 0.06 | 0.25 | 0.04 | 0.12 | 0.2 | 0.02 | 0.02 | 0.03 |
| 2015 | 0.01 | 0.01 | 0.01 | 0.04 | 0.18 | 0.17 | 0.12 | 0.14 | 0.11 | 0.05 | 0.11 | 0.04 |
| 2016 | 0.01 | 0.02 | 0.06 | 0.11 | 0.18 | 0.09 | 0.22 | 0.17 | 0.18 | 0.09 | 0.06 | 0.04 |
| 2017 | 0.02 | 0 | 0.01 | 0.1 | 0.19 | 0.09 | 0.13 | 0.16 | 0.07 | 0.19 | 0 | 0.01 |
| 2018 | 0.01 | 0.02 | 0.05 | 0.06 | 0.15 | 0.24 | 0.2 | 0.12 | 0.22 | 0.09 | 0.03 | 0.06 |
| 2019 | 0.02 | 0.06 | 0.09 | 0.2 | 0.15 | 0.08 | 0.22 | 0.07 | 0.2 | 0.13 | 0.04 | 0.05 |
| 2020 | 0.02 | 0.03 | 0.09 | 0.04 | 0.11 | 0.14 | 0.18 | 0.12 | 0.03 | 0.03 | 0.05 | 0.01 |
| 2021 | 0.03 | 0.03 | 0.07 | 0.05 | 0.09 | 0.02 | 0.04 | 0.15 | 0.17 | 0.11 | 0.02 | 0.02 |
| 2022 | 0.02 | 0.01 | 0.06 | 0.12 | 0.13 | 0.04 | 0.05 | 0.1 | 0.02 | 0.01 | 0.04 | 0.06 |
| 2023 | 0.04 | 0.05 | 0.03 | 0.13 | 0.23 | 0.1 | 0.03 | 0.06 | 0.02 | 0.21 | 0 | 0.08 |
|  |  |  |  |  |  |  |  |  |  |  |  |  |
| Monthly Mean Temperature, Lamberton | | | | | | | | | | | | |
| Year | Jan | Feb | Mar | Apr | May | Jun | Jul | Aug | Sep | Oct | Nov | Dec |
| 2000 | 15.4 | 27.2 | 38.9 | 43.9 | 59.9 | 66.6 | 71.1 | 70.3 | 59.7 | 50.8 | 28.5 | 5.1 |
| 2001 | 18.6 | 8.2 | 24.4 | 45.2 | 58.5 | 68.5 | 73.5 | 70.7 | 59.9 | 47.6 | 45.2 | 25.2 |
| 2002 | 22.9 | 27.3 | 20.8 | 44 | 52.6 | 71 | 75.6 | 68.9 | 62.4 | 40.9 | 32.2 | 25.8 |
| 2003 | 15.2 | 14.3 | 29.1 | 46.7 | 55.3 | 66.2 | 71.3 | 71.2 | 59.6 | 50.9 | 30.1 | 23.6 |
| 2004 | 12.1 | 18.2 | 35.2 | 47 | 55.8 | 64.2 | 69.6 | 63.7 | 65.1 | 50.3 | 37.2 | 22.9 |
| 2005 | 13.9 | 26.1 | 29.9 | 50.4 | 55.4 | 71.5 | 73.3 | 68.6 | 65.9 | 50.4 | 35.9 | 16.6 |
| 2006 | 28.1 | 18.3 | 29.7 | 50.7 | 59.2 | 68.8 | 74.5 | 70.1 | 57.9 | 45.6 | 34.3 | 27.1 |
| 2007 | 18.2 | 11.9 | 35.3 | NA | 62.3 | 70.1 | 73.1 | 69.2 | 62.6 | 53.2 | 33.7 | 14.9 |
| 2008 | 11.5 | 13.3 | 27.4 | 41.9 | 55.9 | 67 | 72.9 | 69.5 | 61.8 | 48.3 | 33.3 | 12.5 |
| 2009 | 7.9 | 19.2 | 28.8 | 44.1 | 58.1 | 66.2 | 67.1 | 67.5 | 64 | 41.5 | 40.7 | 13.6 |
| 2010 | 8.3 | 12.5 | 34.9 | 53.1 | 58.4 | 68.1 | 73.2 | 73.9 | 59.1 | 51.8 | 33.8 | 12.5 |
| 2011 | 6.7 | 14.3 | 25 | 43.6 | 56.5 | 68 | 77.3 | 70.4 | 60.1 | 52.3 | 36 | 25.6 |
| 2012 | 23 | 25.7 | 46 | 49.3 | 62.7 | 70.9 | 78.2 | 68.9 | 60.7 | 45 | 35.1 | 19.6 |
| 2013 | 16.3 | 16.1 | 24.1 | 35.8 | 55.1 | 67.6 | 72.3 | 70 | 65.4 | 47.9 | 30.3 | 10.6 |
| 2014 | 9.1 | 9.3 | 24.6 | 42 | 56.8 | 68.2 | 68.8 | 69.9 | 61.4 | 49.3 | 23.8 | 23.4 |
| 2015 | 18.9 | 10.9 | 34 | 47.5 | 57 | 68.5 | 71.1 | 67.4 | 67 | 50.6 | 39.4 | 26.9 |
| 2016 | 15.5 | 24.1 | 38.6 | 47.5 | 58.5 | 70.5 | 71.8 | 70.5 | 63.9 | 50.1 | 41.7 | 17.2 |
| 2017 | 18.2 | 30.9 | 31.4 | 47.1 | 56.5 | 69.3 | 72.3 | 65.9 | 63.9 | 49 | 31.7 | 19.2 |
| 2018 | 13.1 | 10.8 | 28.1 | 32.1 | 64.5 | 71.1 | 71.9 | 69.8 | 63.5 | 43 | 25.5 | 21.5 |
| 2019 | 10.9 | 4.7 | 23.9 | 44 | 53.3 | 69.1 | 72.8 | 67.5 | 64.4 | 44.1 | 29.1 | 20.2 |
| 2020 | 16.3 | 18.2 | 34.6 | 42.4 | 55.6 | 72.8 | 73.1 | 71.2 | 60.5 | 41.3 | 37.6 | 25 |
| 2021 | 21.9 | 8.1 | 38 | 44.1 | 57.1 | 73.1 | 72.5 | 71.4 | 64.1 | 53.2 | 35.9 | 22.3 |
| 2022 | 9.3 | 13 | 30.5 | 38.4 | 58.2 | 71.2 | 73.7 | 69.7 | 63.2 | 48.8 | 33.4 | 14.2 |
| 2023 | 15.5 | 15.4 | 21.1 | 41.5 | 62.2 | 72 | 69.6 | 71.3 | 66.2 | 49.7 | 36.1 | 32 |
|  |  |  |  |  |  |  |  |  |  |  |  |  |
| Monthly Mean Precipitation, St. Paul | | | | | | | | | | | | |
| Year | Jan | Feb | Mar | Apr | May | Jun | Jul | Aug | Sep | Oct | Nov | Dec |
| 2000 | 0.02 | 0.04 | 0.04 | 0.05 | 0.13 | 0.14 | 0.2 | 0.11 | 0.13 | 0.04 | 0.12 | 0.03 |
| 2001 | 0.04 | 0.03 | 0.03 | 0.26 | 0.16 | 0.14 | 0.06 | 0.1 | 0.11 | 0.05 | 0.1 | 0.02 |
| 2002 | 0.01 | 0.01 | 0.05 | 0.12 | 0.1 | 0.33 | 0.25 | 0.19 | 0.13 | 0.14 | 0 | 0.01 |
| 2003 | 0.01 | 0.02 | 0.04 | 0.08 | 0.22 | 0.22 | 0.07 | 0.03 | 0.07 | 0.03 | 0.02 | 0.01 |
| 2004 | 0.02 | 0.04 | 0.07 | 0.09 | 0.2 | 0.11 | 0.16 | 0.05 | 0.14 | 0.12 | 0.03 | 0.01 |
| 2005 | 0.02 | 0.03 | 0.04 | 0.1 | 0.11 | 0.19 | 0.1 | 0.14 | 0.2 | 0.2 | 0.04 | 0.02 |
| 2006 | 0.02 | 0.01 | 0.04 | 0.15 | 0.1 | 0.14 | 0.08 | 0.28 | 0.1 | 0.02 | 0.03 | 0.06 |
| 2007 | 0.01 | 0.04 | 0.12 | 0.04 | 0.09 | 0.05 | 0.07 | 0.19 | 0.22 | 0.15 | 0 | 0.03 |
| 2008 | 0 | 0.01 | 0.05 | 0.14 | 0.09 | 0.09 | 0.07 | 0.07 | 0.06 | 0.08 | 0.04 | 0.04 |
| 2009 | 0.01 | 0.03 | 0.05 | 0.05 | NA | 0.09 | 0.05 | 0.2 | 0.01 | 0.2 | 0.01 | 0.06 |
| 2010 | 0.02 | 0.03 | 0.02 | 0.06 | 0.08 | 0.21 | 0.18 | 0.23 | 0.17 | 0.05 | 0.07 | 0.1 |
| 2011 | 0.03 | 0.04 | 0.09 | 0.1 | 0.14 | 0.15 | 0.34 | 0.18 | 0.02 | 0.03 | 0.01 | 0.02 |
| 2012 | 0.02 | 0.06 | 0.04 | 0.11 | 0.27 | 0.12 | 0.16 | 0.04 | 0.01 | 0.05 | 0.02 | 0.06 |
| 2013 | 0.02 | 0.05 | 0.06 | 0.17 | 0.23 | 0.24 | 0.1 | 0.05 | 0.04 | 0.14 | 0.02 | 0.04 |
| 2014 | 0.05 | 0.04 | 0.03 | 0.23 | 0.11 | 0.31 | 0.09 | 0.1 | 0.07 | 0.05 | 0.03 | 0.03 |
| 2015 | 0.01 | 0.01 | 0.02 | 0.07 | 0.16 | 0.11 | 0.2 | 0.09 | 0.13 | 0.09 | 0.15 | 0.07 |
| 2016 | 0.01 | 0.03 | 0.07 | 0.12 | 0.07 | 0.12 | 0.19 | 0.32 | 0.17 | 0.11 | 0.09 | 0.06 |
| 2017 | 0.03 | 0.03 | 0.02 | 0.12 | 0.21 | 0.11 | 0.08 | 0.29 | 0.04 | 0.16 | 0.01 | 0.02 |
| 2018 | 0.03 | 0.04 | 0.04 | 0.08 | 0.11 | 0.15 | 0.13 | 0.09 | 0.24 | 0.11 | 0.05 | 0.04 |
| 2019 | 0.01 | 0.08 | 0.07 | 0.11 | 0.21 | 0.1 | 0.15 | 0.22 | 0.16 | 0.16 | 0.06 | 0.08 |
| 2020 | 0.03 | 0.02 | 0.09 | 0.06 | 0.14 | 0.14 | 0.07 | 0.12 | 0.04 | 0.07 | 0.05 | 0.03 |
| 2021 | 0.02 | 0.02 | 0.09 | 0.08 | 0.11 | 0.05 | 0.05 | 0.21 | 0.06 | 0.07 | 0.03 | 0.06 |
| 2022 | 0.02 | 0.02 | 0.1 | 0.12 | 0.16 | 0.03 | 0.04 | 0.15 | 0.01 | 0.01 | 0.07 | 0.06 |
| 2023 | 0.06 | 0.08 | 0.06 | 0.13 | 0.05 | 0.07 | 0.13 | 0.18 | 0.19 | 0.13 | 0 | 0.08 |
|  |  |  |  |  |  |  |  |  |  |  |  |  |
| Monthly Mean Temperature, St. Paul | | | | | | | | | | | | |
| Year | Jan | Feb | Mar | Apr | May | Jun | Jul | Aug | Sep | Oct | Nov | Dec |
| 2000 | 17.1 | 28.7 | 41.8 | 47.9 | 62 | 66.9 | 72.8 | 72.5 | 62.7 | 54.7 | 32.4 | 8.6 |
| 2001 | 21.7 | 13.6 | 29 | 49.1 | 60.8 | 69.4 | 75.2 | 73.8 | 61.5 | 49.5 | 46.9 | 28.1 |
| 2002 | 25.5 | 29.5 | 25.3 | 46.4 | 55.3 | 71.1 | 77 | 71.1 | 65.5 | 43.1 | 33.4 | 27.2 |
| 2003 | 16.2 | 16.8 | 32.4 | 49 | 58.2 | 68 | 72.6 | 74.8 | 63.3 | 52.1 | 33 | 25.9 |
| 2004 | 12.2 | 22.9 | 36.2 | 51 | 57.5 | 65.5 | 71.2 | 66.3 | 67.9 | 51 | 38.6 | 22.7 |
| 2005 | 16 | 27.2 | 32.6 | 52.6 | 56.5 | 73.1 | 75.5 | 71.1 | 67.4 | 53.2 | 37.7 | 20.2 |
| 2006 | 29 | 20.8 | 34.1 | 54.1 | 61.7 | 70.2 | 77.6 | 72.1 | 59.9 | 47.3 | 37.8 | 29.3 |
| 2007 | 20.4 | 13.5 | 39 | 47.3 | 65.1 | 72.3 | 74.7 | 71.8 | 65.8 | 55.4 | 35.2 | 17.1 |
| 2008 | 14.7 | 17 | 29.3 | 45.2 | 56.9 | 67.9 | 74.6 | 71.7 | 64 | 51.3 | 35.3 | 14.4 |
| 2009 | 9.3 | 21.9 | 33.4 | 48 | 58 | 66.1 | 67.4 | 67.5 | 65.3 | 42 | 40.8 | 16.1 |
| 2010 | 11.2 | 17.4 | 37.8 | 53.2 | 59.5 | 66.9 | 73.6 | 74 | 58 | 51.9 | 32.8 | 14.1 |
| 2011 | 9.2 | 15.9 | 26.1 | 44 | 56.2 | 66.6 | 76.3 | 71.1 | 60.7 | 53.1 | 37.1 | 25.4 |
| 2012 | 21.1 | 25.9 | 45.7 | 48.1 | 62.2 | 69.9 | 78.1 | 70.5 | 61.8 | 45.4 | 34.6 | 21.5 |
| 2013 | 14.9 | 15.6 | 23.7 | 37.1 | 54.7 | 66.1 | 71.3 | 71.1 | 64.7 | 47.9 | 30.4 | 10.7 |
| 2014 | 5.3 | 5.6 | 21.3 | 40.3 | 56.8 | 69.9 | 71.3 | 72 | 61 | 47.4 | 23.9 | 23.7 |
| 2015 | 16.5 | 8.9 | 31.9 | 47.3 | 56.9 | 67.7 | 71.4 | 68.2 | 65.9 | 49.6 | 39.8 | 28.2 |
| 2016 | 14.7 | 22 | 38.3 | 45.8 | 58.6 | 68.7 | 72.5 | 70.6 | 63.5 | 50.3 | 42.4 | 19.4 |
| 2017 | 18.5 | 27.9 | 30.7 | 47.2 | 56 | 68.6 | 72.1 | 65.5 | 63.7 | 49.1 | 31 | 17.1 |
| 2018 | 12.9 | 12.2 | 29.5 | 33.5 | 65.3 | 73 | 76.6 | 74.3 | 67 | 44.2 | 25.7 | 23.6 |
| 2019 | 13.1 | 9.4 | 26.1 | 45.5 | 52.8 | 67.2 | 72.5 | 68 | 63.6 | 44.8 | 28.3 | 21 |
| 2020 | 18.1 | 18.1 | 34 | 42.2 | 56.8 | 70.1 | 73.8 | 71.1 | 59.3 | 41.2 | 37.1 | 23.4 |
| 2021 | 19.6 | 9.5 | 37.6 | 44.7 | 57.3 | 73.6 | 73.5 | 72.3 | 63.5 | 53.9 | 34.8 | 21.9 |
| 2022 | 6.2 | 10.8 | 28.4 | 38.6 | 58.6 | 69.7 | 73.4 | 70.1 | 63.4 | 49.6 | 33.7 | 15 |
| 2023 | 19.2 | 18 | 25.7 | 42.7 | 61.2 | 71.9 | 71.2 | 70.8 | 66 | 50.3 | 35.2 | 31.8 |
