# Supplementary material for: Assessment of genomic prediction and genetic gain in multi‑population half-sib families in the perennial grass crop intermediate wheatgrass
Source: PLoS One. 2026 Sep 2;21(9):e0357452. doi: 10.1371/journal.pone.0357452 (PMC13537581; doi:10.1371/journal.pone.0357452)
Supplement: Supplementary file 5 [file pone.0357452.s005.docx]

**S5 Table:** Trait mean and variance in UMN IWG breeding cycles C1-C5 and the half-sib families. Note that C1-C5 yield are per plant basis while that for HSFs and checks is on per hectare basis.

|  | **Mean** | | | **Variance** | | |
| --- | --- | --- | --- | --- | --- | --- |
| **Cycle** | **Yield** | **TKW** | **Height** | **Yield** | **TKW** | **Height** |
| C1 | 64.53 | 8.18 | 134.23 | 1399.13 | 2.11 | 296.73 |
| C2 | 52.96 | 6.87 | 139.98 | 996.65 | 4.44 | 540.66 |
| C3 | 36.43 | 7.92 | 126.68 | 590.75 | 2.82 | 337.76 |
| C4 | 35.36 | 8.27 | 131.98 | 448.31 | 2.19 | 597.33 |
| C5 | 33.31 | 8.33 | 129.47 | 754.77 | 1.83 | 293.42 |
| HSFs | 649.56 | 6.85 | 132.48 | 75442.51 | 0.79 | 207.05 |
| MN-Clearwater (check) | 622.25 | 6.55 | 140.61 | 84994.19 | 1.24 | 74.34 |
| MN1502 (check) | 675.69 | 6.61 | 137.01 | 111432.59 | 1.23 | 85.86 |
